# Diagnosis of Lynch Syndrome and Strategies to Distinguish Lynch-Related Tumors from Sporadic MSI/dMMR Tumors

**DOI:** 10.3390/cancers13030467

**Published:** 2021-01-26

**Authors:** Julie Leclerc, Catherine Vermaut, Marie-Pierre Buisine

**Affiliations:** 1 Molecular Oncogenetics, Department of Biochemistry and Molecular Biology, CHU Lille, F-59000 Lille, France; julie.leclerc@chru-lille.fr (J.L.); catherine.vermaut@chru-lille.fr (C.V.); 2 Univ. Lille, UMR9020-U1277—CANTHER—Cancer Heterogeneity Plasticity and Resistance to Therapies, F-59000 Lille, France; 3 CNRS, UMR9020, F-59000 Lille, France; 4 Inserm, U1277, F-59000 Lille, France

**Keywords:** Lynch syndrome, microsatellite instability, mismatch repair genes, *MLH1* promoter methylation, *BRAF* mutation

## Abstract

**Simple Summary:**

Microsatellite instability (MSI) is a hallmark of Lynch syndrome (LS)-related tumors but is not specific, as most of MSI/mismatch repair-deficient (dMMR) tumors are sporadic. Therefore, the identification of MSI/dMMR requires additional diagnostic tools to identify LS. In this review, we address the hallmarks of LS and present recent advances in diagnostic and screening strategies to identify LS patients. We also discuss the pitfalls associated with current strategies, which should be taken into account in order to improve the diagnosis of LS.

**Abstract:**

Microsatellite instability (MSI) is a hallmark of Lynch syndrome (LS)-related tumors but is not specific to it, as approximately 80% of MSI/mismatch repair-deficient (dMMR) tumors are sporadic. Methods leading to the diagnosis of LS have considerably evolved in recent years and so have tumoral tests for LS screening and for the discrimination of LS-related to MSI-sporadic tumors. In this review, we address the hallmarks of LS, including the clinical, histopathological, and molecular features. We present recent advances in diagnostic and screening strategies to identify LS patients. We also discuss the pitfalls associated with the current strategies, which should be taken into account to improve the diagnosis of LS and avoid inappropriate clinical management.

## 1. Introduction

Microsatellite instability (MSI) is a hallmark of tumors developed in the context of Lynch syndrome (LS). LS is a hereditary cancer syndrome with an estimated population frequency of 1:300 individuals. It is characterized by an increased risk of colorectal cancer (CRC) and endometrial cancer but, also, of ovarian, hepatobiliary tract, upper urinary tract, small bowel, gastric, pancreatic, brain, and skin cancers, more often occurring at a young age [1,2,3,4].

LS is mainly caused by dominant germline inactivating variations in the DNA mismatch repair (MMR) genes *MLH1*, *MSH2*, *MSH6*, or *PMS2*. As a consequence, tumors from LS patients display MSI and a loss of expression of MMR proteins. Diagnosis of LS is important, since patients with LS benefit from adapted protocols of clinical management with surveillance and more radical surgery [5,6,7]. Preventive measures include regular colonoscopic and gynecological surveillance of MMR pathogenic variant carriers, with the frequency and age of initiation dependent on the mutated MMR gene. Patients with LS may also benefit from different approaches for adjuvant therapy [8,9]. However, MSI is not restricted to LS. In fact, only 15–20% of MSI/MMR-deficient (dMMR) tumors can be attributed to LS, and most MSI/dMMR tumors are sporadic. This emphasizes the importance of distinguishing these two entities for appropriate management. This review will address the hallmarks and pitfalls of LS diagnosis. Particular emphasis will be given to the tools and optimal strategies allowing distinction of LS and sporadic MSI tumors.

## 2. Tumor Characteristics in Lynch Syndrome

CRC and endometrial cancer are the most common cancers in LS. The lifetime risk of CRC depends on sex and on the MMR gene, which is mutated. In two recent international prospective studies including more than 3000 families, the lifetime risks of CRC at 70 years old (y.o.) for *MLH1* and *MSH2* gene mutation carriers ranged from 40% to 52%, with a slight male predominance [2,3]. The cumulative lifetime risks for colorectal malignancy were lower in patients with *MSH6* and *PMS2* gene mutations (approximately 15% and between 3% and 13%, respectively) [2,3,10]. Endometrial cancer is the most common extracolonic cancer in LS, with lifetime risk estimates of 35–40% for *MLH1*, 46–53% for *MSH2*, up to 46% for *MSH6*, and 13% for *PMS2*.

The clinical criteria for identifying patients with a high risk of being affected by LS are referred to as the Amsterdam criteria [11] (Table 1), with very high specificity (98%) but low sensitivity (22–42%), as more than 50% of families with LS fail to meet these criteria [12,13,14]. Thereby, the less-stringent Bethesda Guidelines, later updated to the revised Bethesda Guidelines (Table 1), have improved the identification of cases with an older age at onset and/or with no strong family history by selecting the cases that should benefit from a tumor analysis, i.e., microsatellite analysis and/or immunohistochemistry of the MMR proteins [15]. The revised Bethesda Guidelines yielded significantly greater sensitivity compared to the Amsterdam criteria but lower specificity (82–95% and 77–93%, respectively) [12,13,14,16].

It has nevertheless been shown that a non-negligible proportion of patients with cancer linked to LS were not detected with these criteria and that improvement of the screening strategies was needed [16,17]. Thus, current practices tend towards the universal screening of all CRC and endometrial cancers, especially since the result of a tumor analysis can also have an impact on the therapeutic strategy [14,18,19,20,21,22,23,24]. Universal screening is also under consideration for tumors from other organs [25,26].

### 2.1. Histopathology

#### 2.1.1. Colorectal Cancers

More than 75% of sporadic MSI cancers occur in elderly women (mean age at diagnosis 72–74 y.o.), whereas LS-associated CRC tend to occur in younger patients (45–60 y.o.) [27,28,29,30,31], with a slight male predominance [2,29]. Patients with a left-sided or rectal MSI/dMMR tumor are likely to have LS. Indeed, while more than 75% of sporadic MSI cancers occur in the proximal colon, LS-associated CRC have no preferential location, since up to 45% are diagnosed in the left colon and rectum [30]. In their study, Mas-Moya et al. [32] showed that nearly all patients with left-sided or rectal adenocarcinoma (23/24, 96%) demonstrated a germline MMR gene mutation confirming LS.

The features of MSI/dMMR CRC have been well-reported and included in the Bethesda Guidelines. They include poor differentiation, medullary growth pattern, a high mucinous component, signet ring cells, numerous tumor-infiltrating lymphocytes (TILs), and a Crohn’s-like reaction [15,27,33,34,35,36]. Moreover, it was recently reported that programmed death-ligand 1 (PD-L1), which is a major ligand of immunosuppressive receptor PD-1, is frequently expressed at the surface of MSI CRC [37]. These features are typical of MSI tumors, without distinction of LS or sporadic cancer, the latter representing the largest proportion of MSI tumors (around 75%), secondary to hypermethylation of the *MLH1* promoter [16,17] (see Section 4.2.1).

Several studies attempted to identify the morphologic features specific to LS-related tumors, but this led to conflicting results. Some studies demonstrated a tendency towards more frequent poor differentiation [27,30], medullary morphology [29,30], mucinous component [27,33], TILs [29], or PDL1 expression [30] in sporadic MSI CRC vs. LS-associated tumors. In contrast, other studies, including prospective studies [29] and studies with well-defined LS patients (i.e., with a germline mutation), demonstrated no significant difference between sporadic- and LS-related CRC [34,38,39].

#### 2.1.2. Endometrial Cancers

MSI/dMMR endometrial cancers represent 25–30% of endometrial cancers [18,40]. The mean age of onset in LS patients is 47–55 y.o. vs. 62 y.o. in the general population [20,41,42,43]. Like MSI/dMMR CRC, most MSI/dMMR endometrial cancers are sporadic, related to somatic hypermethylation in the *MLH1* promoter [43,44,45,46] (see Section 4.2.1).

The morphological features observed in MSI endometrial carcinomas include the presence of dense peritumoral lymphocytes (PTLs), prominent TILs, and tumor heterogeneity (defined as two morphologically distinct tumor populations), which are features also identified in MSI/dMMR colorectal cancers. The TIL count has a sensitivity of 85% in predicting the MSI status in endometrioid endometrial carcinoma [47,48]. Some other characteristics have been described, such as a tendency to be located at the lower uterine segment, a higher histological grade, myometrial invasion, endometrioid type, isthmic involvement, and dedifferentiation [41,42,48,49,50], but with conflicting results and the absence of a significant association with the MSI status in some of the studies [40,47,51].

Studies investigating the pathological characteristics specific to LS-associated endometrial cancers are limited by the small size of cohorts and the variability in the definition of the controls groups. Tumors in LS are less-often diagnosed at an early stage (57% stage I) [18]. A trend towards more non-endometrioid tumors (particularly, clear cell carcinomas, rare serous carcinomas, and malignant mixed Mullerian tumors or carcinosarcomas) and less mucinous differentiation was observed in LS patients compared to sporadic tumors [42,52,53]. Mills et al. showed that more than half of the LS-related endometrial tumors (58%) did not have MSI tumor features, i.e., lower uterine segment location, tumor heterogeneity, TILs, and PTLs [19]. However, a recent study showed a significantly higher density of infiltrating immune cell effectors in LS-associated endometrial cancers compared to sporadic MMR-deficient endometrial cancers, with more CD8+, CD45RO+, and PD1+ T-cells at the invasive margin [51]. In conclusion, morphologic features are not sensitive enough to detect LS-related endometrial cancers, and universal screening is recommended [18,19,20,54,55].

#### 2.1.3. Ovarian Cancers

MSI/dMMR ovarian cancers are characterized by the early stage at diagnosis (48 y.o. vs. 55–60 y.o. in the general population) [56]. Aysal et al. showed that morphologic criteria such as TILs, PTLs, and dedifferentiated morphology are not sensitive enough to detect MSI/dMMR ovarian cancers, as these features are present in only 14% of the MSI/dMMR ovarian cases [57]. However, dMMR ovarian cancers have been shown to exhibit significantly increased CD3+ and CD8+ TILs and PDL1+ intra-tumoral immune cells [58].

The cumulative lifetime risk of ovarian cancer in LS varies between 6% and 17% [2,3]. The mean age at diagnosis is 43–46 y.o. (vs. 59 y.o. in the general population) [59,60,61,62]. Around 6% of the women are <35 y.o. at diagnosis. LS-related ovarian cancers typically present at lower grades and lower stages compared to sporadic cancers (FIGO (International Federation of Gynecology and Obstetrics) stage I or II in about 65%) [61,63], with better overall survival. The histopathological types of ovarian cancer are mixed type (mucinous/endometrioid/clear cell) (33%), endometrioid (25–40%), serous (21–36%), or clear cell carcinomas (6–17%) [59,60,63,64]. Mucinous or borderline tumors are also described but represent less than 5% of cases.

#### 2.1.4. Urothelial Carcinomas

About 3–12% of upper tract urothelial carcinomas (UTUC) exhibit dMMR, which most commonly manifests as a dual loss of MSH2 and MSH6 or isolated MSH6 loss (up to 86%) [25,65,66,67]. Among tumors with a loss of MMR proteins, LS represents about 20–30% of the cases [65,67,68]. UTUC is the most common urologic malignancy, occurring in 5% of patients with LS [69,70]. Patients with LS are 22 times more likely to develop UTUC than the general population [71] and have a lifetime risk of developing UTUC of up to 15–20%, with the highest risk among patients with *MSH2* mutations (16% vs. 3% to 4% for *MLH1* or *MSH6*) [2].

Some morphologic features have been described in an attempt to discriminate dMMR and MMR-proficient (pMMR) UTUC: increased intra-tumoral lymphocytes, lack of nuclear pleomorphic, inverted growth pattern (or endophytic), and the presence of pushing borders. The inverted growth pattern, at least focally, and increased intra-tumoral lymphocytes have been shown to be predictive of MSI in some studies [66,72], but they did not reach statistical significance in others [25]. Otherwise, there is no significant difference in terms of tumor grade, stage [25], or age at onset, which is comparable to the general population (about 65–70 years old) [65,66,67]. Finally, there are too-few studies with a significant number of genetically characterized patients to identify the characteristics specific to LS. Bladder urothelial cancer as an LS-related cancer is a subject of debate, but a study showed statistically significant differences in their frequencies, with more cancers in *MSH2* mutation carriers (4.4%) compared to *MSH6* (1.7%) or *MLH1* (2.2%) mutation carriers [73]. 

#### 2.1.5. Non-Colorectal Digestive Cancers

The prevalence of MSI/dMMR gastric cancers is estimated at 9–13% [74,75]. MSI/dMMR gastric carcinomas tend to occur as poorly differentiated adenocarcinomas in the antrum of elderly patients and to display abundant T-cell infiltration, intestinal histological type, a lack of lymph node metastases, and TNM (Tumor Node Metastasis) stage I-II [75,76]. In LS, the cumulative risk of developing gastric cancer is estimated to be 2.5–6% [2,4]. LS-associated gastric cancers are mostly of the intestinal type, but the diffuse type is also observed [77]. Helicobacter pylori infection has been reported in about 20% of gastric cancers in LS, with no difference from the general population. The clinical factors associated with the risk of gastric cancer in LS patients are the existence of first-degree relative(s) affected by gastric cancer (odds ratio (OR), 2.52; 95% CI, 1.42–4.45), older age (OR, 2.07 per 10 years; 95% CI, 1.64–2.61), and male sex (OR: 2.82; 95% CI, 1.48–5.38) [78].

Cholangiocarcinomas exhibit MMR deficiency in about 6–10% of the cases. Age, location (intra- vs. extrahepatic), gender, and phenotype (pancreatobiliary vs. intestinal or mixed) are not significantly different between microsatellite stable (MSS) and MSI cases. MSI tumors are significantly more likely to show a solid, mucinous, or signet ring pattern, compared to the typical invasion pattern in MSS tumors (defined as invasive glands without any of the previously mentioned features) [79,80]. In LS, the cumulative risk of cholangiocarcinoma ranges from 0.6% to 3.7% [1,2]. There is a lack of data regarding the characteristics associated with LS-associated cholangiocarcinomas.

Small bowel adenocarcinoma is a rare tumor, about 20% being MSI/dMMR. MSI/dMMR tumors are generally in the duodenum or jejunum, associated with good prognosis, and nonmetastatic [81]. More than 60% of MSI/dMMR small bowel adenocarcinomas may be LS-related according to the family history and MMR protein expression pattern [81]. The lifetime risk for LS patients to develop small bowel cancer is estimated to be 4–12% [2,4,82]. LS-associated tumors are mainly located in the duodenum (43%) and the jejunum (33%), only 7% being located in the ileum [82].

An MMR deficiency is rare in pancreatic cancer, being observed in 1% to 2% of pancreatic ductal adenocarcinomas, with a larger proportion in intraductal papillary mucinous neoplasm (IPMN)-related tumors (about 7%) [83]. MSI/dMMR pancreatic carcinomas show a higher density of CD8+ T-cell lymphocytes at the invasive front compared with MSS/pMMR tumors. The cumulative lifetime risk of pancreatic cancer in LS varies between 0.5% to 3.9%, depending on the mutated gene [2]. LS-associated pancreatic tumors often have a characteristic medullary appearance, with prominent lymphocytic infiltration [84,85]. Pancreatic ductal adenocarcinoma is the predominant tumor type associated with LS [86], even though IPMN have also been reported in LS [87,88,89].

#### 2.1.6. Sebaceous Tumors

Sebaceous tumors, including sebaceoma, sebaceous carcinoma, keratoacanthoma with sebaceous differentiation, and basal cell carcinoma with sebaceous differentiation, are relatively uncommon in the general population [90]. The majority of tumors are solitary and sporadic, with a predilection for the head and neck area of elderly adults [91]. The prevalence of MMR deficiency in sebaceous tumors ranges between 20% and 60%, with a predominance of MSH2 and MSH6 protein loss [92,93,94,95]. Regarding the histopathological characteristics of sebaceous tumors, it has been shown that the number of TILs was increased in dMMR sebaceous neoplasms compared to pMMR sebaceous neoplasms (16.5 vs. 9.7, *p* = 0.027) but with overlaps between both lesions that prevent using TILs as an indicator of the MMR status [93]. A large subset (39–62%) of sebaceous neoplasias is part of Muir-Torre syndrome (MTS), a phenotypic variant of LS characterized by sebaceous neoplasia and at least one visceral malignancy [96]. Sebaceous neoplasms are observed in 5–9% of patients with LS/MTS [97]. The mean age for the first sebaceous neoplasia is about 55 y.o. in MMR mutation carriers, compared to 67 y.o. in patients without a mutation [92,94,98]. The association with LS/MTS is higher for multiple sebaceous tumors, sebaceous adenomas (vs. sebaceous carcinomas), tumors with a keratoacanthoma-like architecture, and an onset before 60 y.o. Although locations outside the head and neck seem more specific to MTS, many cases are described in the head and neck [98]. Sebaceous tumors precede a visceral malignancy diagnosis in 22–60% of cases [99], supporting that the screening of sebaceous neoplasia provides an opportunity to identify LS patients [90,91,92,94,100,101]. To identify LS, a universal routine screening of sebaceous neoplasms has been proposed, regardless of tumor location, age at diagnosis, or other clinical characteristics, especially since sebaceous tumors are rare in the general population [26].

#### 2.1.7. Brain Tumors

The proportion of MSI/dMMR brain cancers is very low (2% to 3%) [8,102]. Some studies focused on these tumors (related to somatic MMR mutations, to LS, or to CMMRD (Constitutional MisMatch Repair Deficiency) syndrome, which is caused by biallelic germline MMR mutations) [103,104] and showed that they can acquire a secondarily proofreading defect due to a mutation in the *POLE* (DNA polymerase epsilon, catalytic subunit) gene, resulting in an ultra-mutant phenotype (>100 mutations/Mb) and a unique mutational signature (“MMR first/POLE second”) [103,104,105]. Additionally, these tumors seem to be more frequently of a particular and very rare histotype characterized by the presence of multinucleated giant cells [106].

Brain tumors are rare in LS, with a cumulative lifetime risk at 70 y.o. estimated to be between 0.5% and 3.7% (the higher risk is for male *MSH2* mutation carriers). However, the question of LS and CMMRD often arises for patients with brain tumors, especially because these tumors often occur at an early age. [2,3,107]. No information is available regarding the specific molecular or phenotypic characteristics of LS- (or CMMRD)-related brain tumors. The histological variant of glioblastoma with multinucleated giant cells is also not specific [106,108,109], but given its rarity (approximately 1% of all glioblastomas), there may be a particular interest in proposing a constitutional MMR gene (and *POLE*) analysis in patients with a giant cell glioblastoma.

#### 2.1.8. Others Cancers

Patients with germline MMR variants can develop various other MSI/dMMR cancers that are not classically linked to LS, such as soft tissue sarcoma, germ cell tumor, prostate cancer, mesothelioma, and melanoma [102].

### 2.2. MSI and Immunohistochemistry in LS

#### 2.2.1. MSI

The molecular hallmark of LS is MSI, which is a consequence of failure of the DNA MMR system that repair errors occurring during DNA replication. Therefore, MSI testing is currently used to evaluate the functionality of the MMR system.

Different panels of microsatellite markers have been used to detect MSI in tumors in order to identify LS. However, two panels have been highly recommended and are widely used, i.e., the “Bethesda panel” (also known as the National Cancer Institute (NCI) panel), which combines two mononucleotide repeats and three dinucleotide repeats [110], and the “pentaplex panel”, which contains five nearly monomorphic mononucleotide markers [111,112]. Compared to the Bethesda panel, the pentaplex panel has been shown to be more sensitive to detect instability (89–96% vs. 76–84%) [21,113,114] and does not require the testing of matching normal DNA [112,115]. Mononucleotide markers also seem more specific to detect LS-associated cancers (specificity about 99%) [113,116] and are thus considered the current standard because of their higher accuracy [21].

The clinical sensitivity of the MSI test for the identification of patients with proven LS varies greatly depending on the mutated gene, the tumor type, and the threshold (i.e., the number of positive markers) used to define a tumor as positive [110,114].

- Gene. The sensitivity of MSI has been shown to be lower in *MSH6* mutation carriers (77% with a pentaplex panel) compared to *MLH1* and *MSH2* mutation carriers (~90%). This is due to fewer unstable markers and a shorter instability length (in bp) observed for MSH6 deficiency [21,114,117,118,119]. In contrast, no particular difficulties have been described for the three other genes, including *PMS2* [120].
- Tumor type. The sensitivity of MSI has been shown to be lower in non-CRC tumors, especially in endometrium, brain, and urothelial tumors, as a consequence of fewer unstable markers and a shorter instability length [25,69,117,118,121,122,123,124,125]. For example, MSI is particularly difficult to detect in brain tumors, with less than 20% of brain tumors from patients with LS (or CMMRD) exhibiting MSI [69,105,126,127,128]. These observations have led to the recommendation to analyze normal DNA in parallel for non-CRC tumors [114,122,124,125].
- MSI-High vs. MSI-Low. Two categories of MSI were initially defined, based on the number of positive markers, i.e., MSI-high (MSI-H), corresponding to an instability at two or more out of the five markers tested, and MSI-low (MSI-L), corresponding to an instability at only one out of the five markers tested) [110,114]. MSI-L colorectal tumors were not shown to differ in their clinicopathologic features or in most molecular features from MSS tumors, leading to consider MSI-L and MSS tumors together and to regard as MSI only MSI-H tumors [129]. However, the sensitivity for LS detection generally increases when MSI-L is considered to be a positive test result (>90% vs. 67–100%) but with lower specificity (45–85% vs. 61–93%) [12,123,130,131,132,133]. As MSI testing is a screening test and not a final diagnostic test, some authors suggest to take into account the MSI-L results to perform constitutional genetic testing [131].

Determination of the MSI status using next-generation sequencing (NGS) and the analysis of sequencing reads at (designated) microsatellite regions has been proposed as an alternative to a Bethesda or pentaplex panel analysis (by a PCR and fragment-length analysis). Although the available data show promising results in detecting MSI tumors in CRC [134], there is a lack of data regarding the performances in other tumors of the LS spectrum, and this requires additional studies [102,135,136].

#### 2.2.2. Immunohistochemistry

Immunohistochemistry (IHC) is also currently used in clinical practice for LS screening. Compared to MSI testing, it allows identification of the putative causative gene/protein. The presence of positive nuclear staining in any tumor cell, with appropriate staining of the internal control cells, is interpreted as a normal expression, whereas a loss of expression with positive internal control cells defines a dMMR tumor. Although some authors suggested to screen tumors only with antibodies against MSH6 and PMS2 proteins (two-stain method) to reduce costs [137,138] (with the subsequent staining of the partner if either is absent), it is generally recommended to test the four MMR proteins, since the two-stain immunohistochemical screening may fail to detect mismatch repair deficiency in some LS tumors [115,139]. For example, staining weaker than the control may be incorrectly interpreted as an intact MMR protein expression, or focal/patchy MSH6 can be retained in the absence of MSH2 [139].

The sensitivity of the IHC test for the identification of patients with proved LS has been estimated to be 83–100%, depending on the studies [17,21,140,141]. However, some expression patterns can lead to misinterpretations. A retained protein expression (diffuse strong intact staining) is well-documented in some cases of *MLH1* mutation or hypermethylation [142,143] but can be found with all four MMR genes. The most frequent cases correspond to pathogenic missense variants associated with nonfunctional protein but retained antigenicity [34,144,145,146], but some cases also involve truncating variations. Certain variants are known to be invariably associated with retained expression of the defective protein, which may lead to the misdiagnosis of LS patients [145,146,147,148]. Approximately 6% of MSI cases escape detection by IHC because of a retained MMR protein expression [146,149]. A weak or partial tumor staining compared to the internal control can also be observed in LS-associated tumors and be a cause of misinterpretation. For example, weak or partial MLH1 staining in *MLH1* mutation carriers was reported in 34% of the tumors by Mangold et al. [143]. Sarode et al. and Watson et al. showed that 42–50% of the cases with indeterminate expressions were found to have the MMR germline mutation, the most frequent being the *MLH1* germline mutation, followed by the *MSH6* mutation [34,150,151]. Thus, weak tumor staining can contribute to missed LS cases when using MMR IHC screening alone. Conversely, preoperative chemotherapy or chemoradiation therapy can be associated with a reduced MSH6 protein expression in the absence of a germline or somatic *MSH6* mutation in MSS CRC, leading to the overestimation of dMMR cancers and LS cases [152,153].

#### 2.2.3. Comparison of MSI and IHC for Identification of LS

The concordance between MSI testing and MMR protein IHC results vary depending on the panel used for MSI and the cancer type but is generally very high, especially for CRC (between 92 and 99%) [17,147,149,150,154,155,156]. A notable exception concerns brain tumors, where IHC has demonstrated its superiority compared to MSI in identifying dMMR glioblastomas but with global limited performances for both technics in these tumors [69,126].

Neither the MSI test nor MMR protein IHC has a sensitivity of 100% for the identification of LS cases. An optimal sensitivity is obtained by combining the two tests, which may accurately identify close to 100% of LS-related tumors. Therefore, a combination of the two techniques should be recommended when possible to increase the chances of LS detection [16,20,115,149].

Finally, it is important to keep in mind that a negative test does not exclude definitively the possibility of LS and that the results should be interpreted with the clinical data, i.e., age at diagnosis and personal and family history of LS-related cancers.

### 2.3. Focus on Colorectal Carcinogenesis and Adenomas in LS

Whereas sporadic MSI CRC arise through the serrated pathway, LS-related CRC arise through the conventional pathway [33]. Some conflicts exist about the natural history of CRC in LS. Classically, MMR deficiency is thought to be acquired during the progression from early to advanced adenomas and to be involved in the acceleration of adenoma progression, since tumor progression in many LS patients takes less than three years, contrasting with a mean of 15 years in the general population [157,158]. This hypothesis suggests that tumor initiation does not depend on MMR implication and that the second gene inactivation hit occurs after the formation of polyps, caused by other events such as *APC* mutations [159]. The correlation of MMR deficiency with a bigger size and higher grade of the adenomas, the presence of more extensive villous architecture, and the prevalence of a subset of small, low-grade MMR-proficient adenomas support the concept of the loss of MMR function as a relatively late event in LS-related CRC [157,160,161].

These findings have been challenged by arguments pointing to the role of MMR mutations in tumor initiation, such as the discovery of a loss of expression of MMR proteins in apparently normal crypts in 25–35% of patients with LS-related CRC [162,163,164]. Indeed, MMR-deficient non-neoplastic intestinal crypt foci (MMR-DCF) were reported to detect LS with excellent specificity (≥99%) [163,164,165]. This implies that MMR protein loss might precede adenoma formation. Furthermore, a high rate of MMR deficiency in LS-associated adenomas (around 70–80%) [157,160,166,167], the complete and homogeneous loss of MMR protein expression in a majority of adenomas, and the rarity of adenomas with a focal loss of MMR proteins (3% in Ahadova et al. [160]) suggest that the MMR system is involved in the initial development of the adenoma as an early event. Nevertheless, it is not clear whether MMR-DCF are cancer precursors in LS, even if MSI was detected in 89% of MMR-DCF [162].

A third pathway, with a direct invasive lesion from the dMMR crypt, could explain the high frequency of interval cancers in patients under regular colonoscopic surveillance [31].

Finally, these conflicting observations could be explained by the existence of multiple tumorigenesis pathways in LS, as proposed by Ahadova et al. [160].

To conclude, even though MMR-DCF detection seems to show an excellent specificity because of the relatively low sensitivity (30–40%) [165], additional studies are needed to demonstrate if it could serve as a biomarker for LS. A study has nevertheless already shown that the sensitivity can be increased with the number of non-neoplastic colonic crypts studied (up to 70% for a median number of colonic crypts analyzed per patient of 3250) [164].

In practice, the combination of MSI and IHC testing in colorectal adenomas allows to screen LS patients with a sensitivity around 70–80% and may be particularly useful when LS is suspected and adenomatous polyps are the only tissues available for analysis [157,160,161,166]. Nevertheless, a negative result does not exclude the presence of LS, especially in the case of low-grade dysplasia adenomas.

## 3. Diagnosis of Lynch Syndrome

### 3.1. Clinical Criteria

The hallmarks of a hereditary cancer syndrome include early age at diagnosis, multiple affected family members, and an increased risk of cancers associated with the syndrome. Accordingly, the usual approach to diagnose LS patients is to use the patients’ personal and family history to guide towards MMR genetic testing.

Several prediction models based on personal and familial clinical data, some including tumor data (MSI and MMR protein IHC), have been developed to evaluate the risk for a given patient to carry a MMR mutation, e.g., MMRpredict [12], PREMM [168], and MMRpro [169]. However, Tresallet et al. evaluated those algorithms in patients with newly diagnosed CRC and showed that the performances of these prediction models were not better than those of the revised Bethesda Guidelines, some LS-patients being missed by all models (elderly patients with no family history) [170]. These results support systematic MSI/dMMR tumoral screening in all CRC.

### 3.2. Molecular Genetic Diagnosis

LS is caused by heterozygous germline inactivating variations in one of the four key DNA MMR genes: *MLH1*, *MSH2*, *MSH6*, and *PMS2*. In 2015, *MLH1*, *MSH2*, *MSH6*, and *PMS2* accounted for 40%, 34%, 18%, and 8%, respectively, of the 3000 unique germline sequence variants of MMR genes deposited in the International Society for Gastrointestinal and Hereditary Tumours (InSiGHT) database [171]. Point mutations are predominant, but large rearrangements can also be the case of LS, representing 10% of the variants in *MSH2* and *PMS2*, 7% in *MLH1*, and 2% in *MSH6* [171]. Variants of unknown significance (VUS) or class 3 variants account for about 20–30% of the MMR variants identified in patients suspected of having LS. For these patients, in vitro MMR functional assays are an important tool to assess the pathogenicity of these variants [172]. Constitutional epimutations of *MLH1* and *MSH2*, characterized by the hypermethylation of the promoter of these genes in normal tissues, are an alternative cause of LS [173,174,175].

The diagnosis of LS is based on the detection of a germline pathogenic mutation or epimutation in a MMR gene. A tumor analysis, i.e., MSI/dMMR status +/− *BRAF* mutational/*MLH1* methylation status, is of great importance to identify the patients who should benefit from MMR germline genetic testing.

Targeted NGS has progressively replaced Sanger sequencing for the molecular diagnosis of LS in most clinical labs, providing multigene panel testing and cost-effectiveness [176]. This strategy can reliably detect nucleotide substitutions and indel within exons and flanking intronic regions and can also detect copy number variations (CNV) using dedicated algorithms. Multiplex ligation-dependent probe amplification (MLPA, MRC-Holland) can be further added to targeted NGS for the detection or the validation of CNV. An analysis of the *PMS2* gene is technically challenging due to the presence of highly conserved pseudogenes that complicate the mutation and CNV detection. Strategies to circumvent this issue include long-range PCR followed by nested PCR and Sanger sequencing or by NGS, cDNA sequencing, MLPA adapted to the potential gene conversion between *PMS2* and the pseudogene *PMS2CL* in the 3’ exons, and NGS with adapted read alignment and variants calling for exons 12–15 [177,178,179,180,181,182]. Regarding epimutations, their detection requires specifically looking for them with dedicated technics.

**Figure 1:**
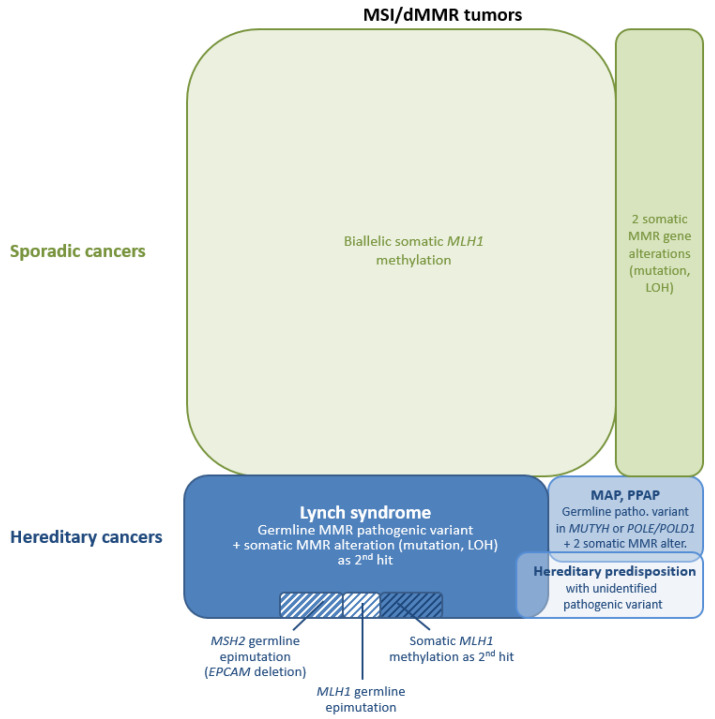
Schematic representation of the mechanisms of mismatch repair (MMR) gene inactivation in Lynch syndrome (LS)-related and sporadic microsatellite instability/mismatch repair-deficient (MSI/dMMR) tumors. Some of these mechanisms explain a potential “misinterpretation” for LS prediction and require dedicated technics. Abbreviations: LOH, loss of heterozygosity; MAP, MUTYH-associated polyposis; patho., pathogenic; and PPAP, polymerase proofreading-associated polyposis.

**Pitfalls**: For a substantial proportion of patients with a MSI/dMMR tumors, even those with early onset CRC [183], no germline mutation is detected in the four MMR genes. In these cases, the MSI/dMMR tumor can be the consequence of an undiagnosed hereditary predisposition to cancer or can be a sporadic tumor. Below are the potential origins of MSI/dMMR tumors without germline MMR variations (Figure 1):

(1) One potential explanation is **a germline alteration in the MMR gene that cannot be detected or is difficult to detect using conventional technics**. Some of these alterations that require specific methods are well-known, and their detection can be implemented in routine diagnosis. Shortly after the description of germline deletions of the last exon(s) of the *EPCAM* gene, leading to epigenetic silencing of the downstream gene *MSH2* in epithelial cells [184], it was acknowledged that 3′-end *EPCAM* deletions can be a frequent cause of unexplained MSH2 deficiency [185,186], and *EPCAM* deletion detection is now included in the routine genetic diagnosis of LS. These deletions are detected by MLPA, as probes at the 3′-end of *EPCAM* are included in MLH1/MSH2-P003 and MSH6-P072 kits. They can also be detected by applying CNV detection-specific algorithms to NGS data if *EPCAM* is included in the multigene panel. Germline epimutations of the *MLH1* gene (i.e., constitutional hypermethylation of the *MLH1* promoter) are occasionally responsible for the MMR-deficient phenotype in LS patients, and their detection is now part of the genetic diagnosis strategy of LS. It requires dedicated technics, which can be based on bisulfite conversion of the DNA (pyrosequencing or (quantitative) methyl-specific PCR) or on methylation-sensitive restriction enzymes (MS-MLPA) [187]. Additionally, highly sensitive technics are required due to the low level of methylation observed at *MLH1* promoter region in peripheral blood mononuclear cell (PBMC) DNA for some patients with a constitutional *MLH1* epimutation [175,188] (see, also, Table S4 in [189]). It should be noted that these technics are not used for *MSH2* epimutation detection, since the methylation of *MSH2* depends on the expression of the *EPCAM* gene and is consequently mostly detected in epithelial tissues where *EPCAM* expression is high and not in PBMC.

Other germline alterations require dedicated technics or the extension of existing methods and are not yet part of the routine diagnostic of LS. Some variations may be located within the promoter, regulatory, or deep-intronic regions of the MMR genes, regions that are not commonly analyzed in routine diagnosis procedures. Moreover, even if detected, these variations are still very difficult to interpret, and their clinical significance remains mostly nonconclusive [190]. There are only a few reports of deep-intronic variants with pathogenic significance. A variation located deep within intron 1 of the *MSH2* gene (NM_000251.3:c.212-478T>G) has been well-documented in a family with LS and is now considered as pathogenic [191]. The effect on splicing of a variation located within *MLH1* intron 15 (NM_000249.4:c.1732-264A>T) has been recently demonstrated [192].

Complex structural rearrangements, especially CNV-neutral ones such as gene inversions and large intronic insertions, are usually not detected with targeted NGS, since their breakpoints are not covered. Paracentric inversions involving *MLH1* [190,193] or *MSH2* genes [194,195,196] have been described as a disease-causing mechanism in LS. A balanced translocation disrupting *MLH1* has been described in a patient from Iceland [197]. The insertion of a 2.2-kb-long retrotransposon in *PMS2* intron 7 has also been described [198]. Morak et al. investigated the prevalence in the German population of five founder CNV-neutral structural variants (SV) and identified only one patient with an insertion in *PMS2* intron 7, concluding that these SV are not frequent [199]. The detection of CNV-neutral SV requires specific methods, such as full-length cDNA or deep-intronic sequencing [190,200], and the optimal strategy is different depending on the type of SV. As the paracentric inversion involving *MSH2* exons 1 to 7 can be a frequent cause of unexplained LS in some populations [201], two specific probes have been added to the commercialized MLPA-kit MLH1/MSH2-P003 (MRC Holland) to detect the rearrangement breakpoint reported in intron 7 [194,195]. However only this specific inversion is detected with these probes, and paracentric inversions involving other regions of *MSH2* are not [196]. Insertions of *Alu* elements, such as the ones reported in the coding sequence of *MSH2* [202] or *MLH1* [188,203], are another type of SV that are not easily detected.

Only a limited number of patients with somatic mosaicism of a MMR gene have been described so far [204,205,206], and the implementation of NGS has not drastically increased the number of these observations. Anyway, analysis pipelines dedicated to mosaic detection in NGS data may be useful for a few patients with no history of cancer in the ascendants, at least to exclude somatic mosaicism. Alternatively, MMR analysis in the tumor and subsequent screen for the pathogenic variants in nontumoral tissues with highly sensitive technics might be the strategy to exclude low-level mosaicism and mosaicism not detectable in lymphocyte DNA [207].

An unidentified germline MMR gene variant remains highly suspected in families with an aggregation of tumors of the LS spectrum, especially when these tumors harbor the same dMMR phenotype.

(2) **Germline pathogenic variants in genes other than the four key MMR genes** can mimic LS. The variable phenotype of MUTYH-associated polyposis can overlap the LS phenotype [208], and biallelic *MUTYH* germline variants impairing the base-excision repair (BER) pathway can sometimes lead to biallelic somatic variants in MMR genes and to MSI/dMMR tumors [209,210,211]. Colorectal tumors from patients with a germline pathogenic *POLE* variant (i.e., variant leading to loss of the proofreading function of the polymerase) are usually MSS [212], but germline variants in *POLE* were sometimes reported in patients with early-onset MSI/dMMR CRC and somatic variants in MMR genes [213]. Mutations of these genes must be considered for patients with MSI/dMMR tumors and cancer-affected relatives, especially when diagnosed with cancer at a young age. These genes are now included in most of the multigene panels analyzed for LS diagnosis.

Other candidate genes with potentially pathogenic germline variants reported in patients with MSI/dMMR tumors (without germline MMR gene mutations) include *MSH3*, *EXO1, FAN1*, *MLH3*, *POLD1*, *RFC1*, *RPA1, SETD2*, *BUB1*, *BARD1*, *WRN*, *MCPH1*, and *REV3L* [214,215,216,217,218]. *MSH3* biallelic variants leading to MSH3 deficiency are associated with the microsatellite instability of di- and tetranucleotides (EMAST, Elevated Microsatellite instability at Selected Tetranucleotide Repeats) [214] but the stability of mononucleotide repeats. Recently, germline biallelic *MCM8* variants have been reported as the cause of MSI in a patient with an early-onset CRC with somatic biallelic variants in *MLH1* [219].

(3) MMR deficiency can also be observed in sporadic tumors, as **the consequence of somatic biallelic inactivation of an MMR gene**. The best-known cause of somatic inactivation leading to sporadic tumors is the epigenetic silencing of the *MLH1* gene, which is responsible for a large proportion of MLH1-deficient tumors, especially in older patients [16,17,43,44,45,46,220].

The coexistence of a pathogenic variant on each allele of an MMR gene or of one pathogenic variant with the loss of the second allele (i.e., loss of heterozygosity, LOH) can also be the cause of MSI/dMMR. Depending on the studies, the proportion of unexplained MSI/dMMR colorectal and endometrial tumors (i.e., tumors without a pathogenic germline variant or somatic *MLH1* promoter methylation), which are caused by biallelic somatic genetic alterations can range from 17% to 95% [205,220,221,222,223,224,225]. It has been shown that, for 25% of unexplained MSI endometrial cancers, a somatic variant in *POLE* could be the cause of these somatic MMR gene alterations [226]. Biallelic somatic MMR inactivation has also been described as a frequent cause of MSI/dMMR sebaceous neoplasms [227]. It should be noted that, unlike tumors with *MLH1* epigenetic silencing, tumors with somatic MMR genetic alterations are not diagnosed at a significantly higher age than those from LS patients [220,223].

Patients with inflammatory bowel diseases are at increased risk of developing sporadic CRC, with about 15% of these tumors exhibiting MSI [228]. Other conditions, such as Hodgkin’s lymphoma treated with radiotherapy or procarbazine-containing chemotherapy, can increase the frequency of MSI CRC with somatic MMR gene alterations [229].

When no germline MMR variant has been detected, somatic MMR testing significantly reduces the number of patients considered to have a LS and to require lifelong Lynch surveillance protocols. Only a limited number of these patients are concerned by an inherited predisposition to cancer and have a dMMR tumor secondary to germline defects in genes other than the MMR genes (see (2)) or to somatic mosaicism in an MMR gene (see (1)). Most of these cases are truly sporadic and, consequently, nonheritable and should therefore be identified to prevent relatives from unnecessary anxiety and colonoscopies and other surveillance procedures. Even if not yet implemented in the recommendations for routine diagnostics, somatic MMR genetic testing should be offered to patients with a MSI/dMMR tumor and negative germline testing. In our routine practice, we propose tumoral MMR genetic testing to these patients when the tumor occurs below 60 y.o. A recent study evaluated that nonheritable causes of MSI/dMMR are eight-fold more common than LS in patients with CRC diagnosed between age 60 and 70 and 20-fold more common in patients between age 65 and 70 [220]. Consequently, above 60 y.o., a sporadic tumor is the most likely explanation when no germline variant has been identified with the current diagnosis procedures, and this does not require further tumor testing.

(4) **False-positives or the misinterpretation of MSI/IHC test results** can be as high as 19% of the unexplained dMMR tumors in some studies [225]. In most cases, MSI testing and immunochemistry showed discordant results, highlighting the need to perform both the test for LS screening and to repeat the test to confirm the discordant results. Before gene panel testing was the standard for a Lynch diagnosis, misleading IHC results leading to selective analysis of the wrong MMR gene could also be a cause of undetected variations [190].

(5) There may also be some **other cellular mechanisms leading to MSI in tumoral cells that could be further investigated** in order to explain MSI tumors without defects in MMR genes. For example, it has been shown that cells lacking the H3K36 trimethyltransferase SETD2 (SET domain containing 2) display MSI owing to the loss of an epigenetic histone mark that is essential for the recruitment of the MSH2-MSH6 complex [230]. It has also been shown that the phosphorylation of PCNA (Proliferating Cell Nuclear Antigen) can inhibit MMR function [231].

The term Lynch-like syndrome (LLS) has been proposed for the patients who present with an MSI/dMMR tumor without *MLH1* promoter hypermethylation when no pathogenic germline MMR gene variation is found. Buchanan et al. combined the results of three different colorectal cancer cohorts and determined that LLS represents 59% (95% CI: 55–64%) of the patients with a MSI/dMMR CRC tested for germline MMR gene variations (433 patients in total after the exclusion of tumors with *MLH1* hypermethylation) [232]. They also combined the results of four endometrial cancer cohorts, where LLS represented 52% (95% CI: 41–62%) of the patients with a dMMR endometrial cancer tested for germline MMR gene variations (101 patients in total after the exclusion of tumors with *MLH1* hypermethylation). Comparable values, ranging from 44% to 61%, were reported in other studies, including more recent ones [222,233,234,235,236].

As detailed above, there are different explanations for the absence of germline MMR gene variant detection, and some patients with LLS have cancers of hereditary origin, whereas others have cancers of sporadic origin. A few studies investigated the risk of colorectal and extracolonic LS-associated cancers in LLS patients and relatives and identified a lower risk compared to LS and a higher risk compared to sporadic cancer or to the general population [4,237,238]. However, in these studies, the proportion of cases explained by biallelic somatic variations in MMR genes was not determined, and LLS patients were considered as a single group, even if most likely heterogeneous. Similarly, variable mean ages at onset, between 48.8 y.o. and 65 y.o., were found for LLS cases, depending on the studies [32,222,236,237,238], and that might be due to variable proportions of undiagnosed germline mutations in these studies.

As our ability to identify genetic variations of various types increases, the number of patients with unexplained MSI/dMMR tumors, i.e., Lynch-like patients, should decrease. Patients with *EPCAM* deletions illustrate this, since they were considered as Lynch-like before this mechanism of *MSH2* inactivation was discovered. Similarly, patients with MSI/dMMR tumors explained by somatic mechanism should not be considered as Lynch-like patients, and patients with documented biallelic somatic variations should probably be excluded from this group, just as patients with biallelic somatic *MLH1* hypermethylation are. Thus, the precise definition of the different groups of patients with MSI/dMMR tumors has major implications on clinical management and genetic counseling.

## 4. Differential Diagnosis of LS-Related vs. Sporadic MSI/dMMR Tumors

Around 13% of all CRC and 20–30% of all endometrial carcinomas are MSI/dMMR. While the sensitivity of MSI/dMMR to predict LS is high, its specificity is low. Indeed, nearly all CRC and most extracolonic tumors from LS patients are MSI/dMMR, but only 22% of MSI/dMMR CRC [17,43] and 8–14% of MSI/dMMR endometrial cancers [20,43,45] harbor MMR germline mutations, requiring additional tools to identify LS (Table 2).

### 4.1. Clinical Presentation

Several clinical features are indicative of LS-related MSI/dMMR cancers:

(1) First, **personal and family histories of LS-related cancers** are strong indicators of LS [20,27,59,233,239,243,244]. Synchronous LS-related tumors are also indicative of LS. The identification of a few additional colorectal polyps at the time of CRC diagnosis is suggestive of LS [239]. Multiple sebaceous neoplasias are a strong indicator of LS/Muir-Torre syndrome [92,94,100,244]. On the other hand, synchronous primary carcinomas of the ovary and endometrium are unlikely to be part of LS [57,245,246]. Certain types of MSI/dMMR cancers are more predictive of LS, e.g., small-bowel adenocarcinoma [233]. More than 60% of MSI/dMMR small-bowel adenocarcinomas may be LS-related [81].

(2) Second, **the age of cancer onset**, which is much lower in patients with LS. Overall, the mean age of diagnosis is more than 10 years earlier than that of sporadic MSI/dMMR cases (48 y.o. vs. 60.5 y.o.) [239]. For example, ovarian cancers occur at a mean age of 45 y.o., i.e., about 20 years earlier than the sporadic cancers, with more than 60% (62–85%) of the tumors diagnosed before 50 y.o. compared to 13% in sporadic cases and about 90% diagnosed before 60 y.o. [59,60,63]. Therefore, an onset before the age of 50–60 y.o. for a LS-associated type of tumor is a strong indicator of LS. However, about 50% of CRC are diagnosed at an age older than 50 y.o. and 17% after 60 y.o. [16,17], and LS women frequently present with endometrial cancer at an age older than 50 y.o. (50–90%, depending on the studies) or even 60 y.o. (15–45%) [20,43,44,45]). Similarly, 25% of patients with proven LS/Muir-Torre syndrome present their first sebaceous tumor after 60 y.o. [92]. Moreover, the mean age at cancer diagnosis is much higher in *MSH6* mutation carriers (about 56 y.o. for CRC and 55 y.o. for endometrial cancer) compared to *MLH1*, *MSH2*, and *PMS2* mutation carriers [119,120,247]. Therefore, an early onset is a strong indicator of LS, but LS cannot be ruled out by an older age at diagnosis.

(3) Third, **the gender and tumor location for CRC**, sporadic MSI CRC being significantly more frequent in elderly women and located in the proximal colon, while LS-related CRC are observed in both genders and occur at equivalent frequencies in the proximal colon and in the distal colon and rectum [27]. Consequently, patients with left-sided or rectal tumor locations are more likely to have LS [32].

(4) The **body mass index** (BMI) is also an indicator for patients with endometrial cancer. Given that obesity is a known risk factor of sporadic endometrial cancer, a BMI within the normal range may support the hypothesis of LS [45,239].

(5) Finally, several **histopathological features** are commonly observed in MSI/dMMR tumors, which include a poor differentiation (for CRC) or over-representation of some histotypes (for CRC, endometrium, and ovary) and prominent TILS and PTLs [33,42,47,50,53,56,57,248,249], but these do not discriminate LS-related and sporadic tumors. Some features may be more specific to LS, such as the presence of conventional adenomas in CRC and of MMR-deficient colonic crypts in peritumoral mucosa [162,163,164] and an intra-tumoral immunological pattern with much higher CD8+ immune cells in endometrial carcinomas [51], which may be helpful for the identification of LS.

### 4.2. Molecular Presentation

LS-related and sporadic MSI/dMMR tumors develop through different mechanisms of MMR gene inactivation. As a consequence, sporadic MSI/dMMR tumors display unique molecular features, which are routinely used to distinguish them from LS-related tumors.

Most of the sporadic MSI/dMMR tumors are due to inactivation of the *MLH1* gene by methylation of its promoter. An immunohistochemistry analysis of the MMR proteins is thus the first step in discriminating LS and sporadic MSI/dMMR tumors, since the combined loss of MSH2 and MSH6 or the isolated loss of MSH6 or PMS2 will argue for a LS-related tumor. In contrast, the detection of MLH1 protein loss is not specific and requires a complementary analysis to distinguish a LS-related tumor caused by a *MLH1* constitutional alteration from a sporadic tumor caused by acquired somatic hypermethylation of the *MLH1* promoter. Of note, zonal heterogeneity of the staining with areas of the tumor showing an abrupt loss of MLH1 and PMS2 protein expression may be an indicator of acquired inactivating alterations of *MLH1* (primarily, *MLH1* promoter hypermethylation), arguing for sporadic cancer rather than LS-related cancer [250,251]. The isolated loss of PMS2 can also be due to *MLH1* promoter hypermethylation [240].

While this reasoning is applicable to the majority of tumors, a notable exception is sebaceous neoplasia. Indeed, most of sebaceous MSI/dMMR tumors display a loss of MSH2 and MSH6 proteins, which is widely considered as highly predictive of LS, but a *MSH2* germline mutation is detected in only one-third of cases [92,94,244], suggesting that a significant proportion of MSI/dMMR sebaceous tumors are sporadic. Accordingly, biallelic somatic MMR inactivation has been described as a frequent cause of MSI/dMMR in sebaceous neoplasms [227].

#### 4.2.1. MLH1 Promoter Methylation

As mentioned above, sporadic MSI/dMMR tumors are generally associated with hypermethylation of the *MLH1* promoter, which coincides with the CpG island methylator phenotype (CIMP) [252,253]. CpG island methylation is a physiological process related to aging, which concerns a number of specific genes in cancer, among which is *MLH1*, leading to their inactivation [253]. In contrast, methylation of the *MLH1* promoter is rare in LS. A *MLH1* promoter analysis in tumors displaying loss of the MLH1 protein is thus very helpful in the distinction of LS-related and sporadic tumors. Importantly, only the methylation status of the proximal region of the promoter is of interest. Indeed, only hypermethylation in the proximal region (usually referred to as regions C and D) correlates with an absence of MLH1 protein expression, region C being the most specific [254,255].

About 75% of MSI and MLH1-deficient CRC unselected for age at diagnosis or a family history of cancer display *MLH1* promoter hypermethylation, the proportion of tumors with *MLH1* promoter hypermethylation increasing with age [16,17,220]. This proportion ranged from 0% in MSI/dMMR CRC below 40 y.o. to 84% in MSI/dMMR CRC between 65 and 69 y.o. in the large prospective cohort reported by Vos et al. [220]. Conversely, hypermethylation of the *MLH1* promoter is uncommon in LS, occurring in less than 6% of tumors from *MLH1* mutation carriers (for review, see [242]). Unmethylated *MLH1* has a sensitivity of 94% and a specificity of 88% for the identification of *MLH1* mutation carriers in patients with a MSI/dMMR CRC with a loss of MLH1 [241].

Regarding endometrial carcinomas, 75–95% of MSI and MLH1-deficient tumors display *MLH1* promoter hypermethylation [43,44,45,46]. As in CCR, hypermethylation of the *MLH1* promoter is rare in LS [45]. Similarly, most of MSI/dMMR gastric cancers, ovarian cancers, and ampullary carcinomas with a loss of MLH1 expression are associated with *MLH1* promoter hypermethylation, suggesting sporadic cancers [56,76,256,257].

#### 4.2.2. BRAF Mutations

Eight–ten percent of all CRC display a somatic mutation in the *BRAF* (MIM*164757) gene. Most of *BRAF* mutations correspond to the NM_004333.4:c.1799T>A, p.Val600Glu (V600E) (78%), which activates the MAPK pathway [258]. This mutation is tightly associated with MSI and *MLH1* promoter hypermethylation in CRC [252]. The BRAF V600E mutation may have a role in the initiation and promotion of colorectal tumorigenesis through the serrated neoplasia pathway [259,260,261]. About 60% (40–73%) of sporadic MSI/dMMR CRC exhibit the BRAF V600E mutation. In contrast, the BRAF V600E mutation is almost never detected in LS-related CRC (1.4%) [241,262,263,264,265,266,267]. Consequently, for tumors with a loss of the MLH1 protein, the detection of a BRAF V600E mutation is a strong negative predictor of LS. The absence of a BRAF V600E mutation has a sensitivity of 98.6% but a specificity of 66% for the identification of *MLH1* mutation carriers in patients with a MSI/dMMR CRC with a loss of MLH1 [241]. Of note, no information is available about non-V600E mutations, which account for about 20% of all BRAF mutations identified in CRC. However, these mutations are uncommon in MSI CRC (6%) and seem to represent a distinct (biologically and clinically) category of tumor [258,268].

*BRAF* mutations are very rare in endometrial cancer (<1%) [44,269,270,271,272], with no association with *MLH1* promoter methylation or MSI/dMMR [269,270]. Only one study identified a relatively high frequency of *BRAF* mutations in endometrial cancers (21%), with an apparent overrepresentation in MLH1-deficient tumors (41% vs. 11% in pMMR tumors) [273]. However, these results are difficult to interpret, as most of these mutations were non-V600E. Anyway, the clinical interest of *BRAF* mutation analysis in endometrial cancer is very unlikely.

*BRAF* mutations are rare in small-bowel adenocarcinoma (7.6%), only 10% of the observed mutations corresponding to V600E and a majority of the others being inactivating mutations [274]. No information is available about the association between BRAF mutations and MSI. Oncogenic *BRAF* mutations are very rare in ovarian cancer (<1%), gastric cancer (1%), cholangiocarcinoma (3%), pancreatic cancer (2%), urothelial carcinoma (4%), and glioblastoma (2%), most of these mutations being non-V600E, except in glioblastoma [62] (Cbioportal: www.cbioportal.org). No *BRAF* mutation has been observed in sebaceous neoplasms from proven or potential LS [275]. Therefore, a *BRAF* mutation analysis in non-CRC tumors has no utility to distinguish sporadic from LS-related tumors.

BRAF V600E IHC has been proposed as an alternative to the *BRAF* mutation molecular analysis, with very variable results. Several studies suggested a very high sensitivity and high specificity of V600E-specific IHC for the detection of *BRAF*-mutated CRC [276,277,278] (sensitivity, 100% and specificity, 98.8–100%), while others pointed out insufficient sensitivity, specificity, and robustness for the use in clinical practice (sensitivity, 51–85% and specificity 68% [279] and sensitivity, 35–71% and specificity, 74% [280]). Anyway, BRAF IHC does not seem robust enough to be currently used as an alternative to the *BRAF* molecular analysis.

#### 4.2.3. BRAF Mutation Analysis vs. MLH1 Methylation Analysis

*BRAF* mutation testing is more common than methylation testing in clinical laboratories, since it is commonly used for therapeutic purposes. Moreover, it is technically easier than a methylation analysis. However, the clinical utility of *BRAF* mutation detection for LS screening is limited, as it is restricted to CRC. Moreover, *BRAF* mutations concern only about 60% of sporadic MSI/dMMR CRC, which implies that about 40% will necessitate a subsequent *MLH1* methylation analysis.

A methylation analysis is technically more challenging than a *BRAF* mutation analysis (and attention must be paid to the region analyzed). However, the clinical value of the *MLH1* promoter methylation analysis is much higher for the identification of *MLH1* mutation carriers among patients with CRC [241,242] and is also applicable to endometrial cancers and probably to other tumor types.

Several cost-effective strategies have been proposed to optimize the identification of LS among MSI/dMMR CRC with a loss of MLH1: (1) a *BRAF* mutation analysis alone, (2) a *MLH1* methylation analysis alone, and (3) a *BRAF* mutation analysis followed by a *MLH1* methylation analysis in cases with no *BRAF* mutation. A *MLH1* hypermethylation analysis does not only outperform a *BRAF* mutation analysis but is also more cost-effective. Using a *BRAF* mutation analysis as a sole test would increase the referral rates for genetic testing by two-to-three-fold compared with a methylation analysis of *MLH1* alone. However, the hybrid approach may facilitate the wider implementation of LS screening without significantly increasing the cost [281,282,283].

**Pitfalls**: Although *MLH1* promoter hypermethylation and BRAF V600E mutation are strong negative predictors for LS, the data must be interpreted with caution.

Regarding *MLH1* promoter hypermethylation, although its negative prediction value for LS is high, the detection of *MLH1* hypermethylation does not exclude a diagnosis of LS. Indeed, *MLH1* promoter hypermethylation may occasionally be the cause of inactivation of the second allele at the somatic level (second hit) in patients with a germline *MLH1* mutation [263,284,285]. A quantitative *MLH1* methylation analysis has been proposed as a mean to distinguish biallelic and monoallelic methylation [286], but this implies an accurate evaluation of the percentage of tumor cells in the sample, which is challenging in a number of cases. Moreover, *MLH1* constitutional epimutation can be the cause of LS [173,175,187]. Similarly, *BRAF* V600E mutations have been occasionally detected in CRC from proven MMR mutation carriers [187,241,262]. Of note, *BRAF* mutations may be more common in CRC from patients with *MLH1* constitutional epimutation (3/30 cases identified with an *MLH1* epimutation [187]) compared to CRC from LS patients with a classical MMR gene mutation. Although rare, one should be aware that the use of *BRAF* mutation and *MLH1* methylation testing to rule out the diagnosis of LS will ignore a few individuals with LS. Moreover, patients with primary *MLH1* epimutation generally do not have a family history of cancer. Therefore, germline MMR mutation testing should still be considered in patients with clinical features highly suggestive of LS, and *MLH1* epimutation testing should be performed in those with a cancer onset below the age of 60 y.o. and a *MLH1*-methylated tumor, irrespective of the *BRAF* mutation status and of the family history.

Conversely, the absence of *MLH1* promoter hypermethylation does not always mean that the patient is affected by LS, since MSI/dMMR tumors may alternatively be the consequence of two somatic mutations or of mutations in another gene than the MMR ones (see Section 3). Such pitfalls underline the importance of interpreting molecular data cautiously.

#### 4.2.4. Other Potential Markers

Other markers have been evaluated for their ability to identify LS, some of which showed a potential interest in the distinction of LS and sporadic MSI/dMMR cancers.

Sporadic CRC with MSI and the hypermethylation of *MLH1* belong to the group of CIMP tumors [253]. *CDKN2A* (*p16*) is among the most frequently methylated loci [253,287,288]. The loss of p16 expression has been shown to be present in 30% of tumors with *MLH1* promoter hypermethylation but in none of the tumors from patients with LS, suggesting that p16 immunochemistry may be used as a surrogate marker for *MLH1* hypermethylation [289,290].

Certain proteins, such as ANXA10 (Annexin A10), are highly expressed in colorectal tumors, arising through the serrated neoplasia pathway [291]. The expression of the ANXA10 protein is observed in about 45% (42–49%) of sporadic CRC, irrespective of the presence of a *BRAF* mutation, but in a low proportion of LS-related CRC (5–12%), suggesting that ANXA10 immunohistochemistry may be used as a supportive marker in association with *BRAF* to distinguish LS and sporadic MSI/dMMR CRC [291,292].

*PIK3CA* mutations may have be interesting for identifying tumors with two somatic MMR gene mutations, excluding a diagnosis of LS. Indeed, *PIK3CA* mutations have been shown to be more frequent in CRC with somatic mutations (67% (14/21)) compared to LS-associated CRC (22% (4/18)) (and CRC with *MLH1* hypermethylation, 20% (2/10)) [293]. Mutations in *PIK3CA* were also present in all endometrial cancers with somatic mutations in the MMR genes tested (*n* = 13) [293]. However, additional studies are needed to evaluate the potential utility of this test in discriminating sporadic and LS-related tumors.

Recently, tumor mutational signatures that provide insights in the etiology of the tumorigenesis processes have been identified [294]. Signature 6 is strongly associated with MSI/MMR deficiency in CRC, but several other signatures have been associated with MMR deficiency in a variety of cancers [294,295]. Signatures 6 and 15 have been shown to be more prevalent in MMR-deficient sebaceous tumors from patients with LS compared with MMR-proficient tumors [296]. However, whether these tumor mutational signatures are able to distinguish LS-related from sporadic MSI/dMMR tumors is currently unknown.

#### 4.2.5. MMR Gene Analysis in Tumors

The risk of cancer in families without a germline mutation has been shown to be lower than the one in families with proven LS but higher than the risk of cancer in families with sporadic cancer and in the general population [4,237,238], justifying surveillance procedures. However, it is now clear that a majority of cases are due to acquired biallelic somatic mutations. Thus, identifying the cause of MSI/dMMR is crucial to guide the clinical management of patients and their families. Further analysis of the four key MMR genes in the tumor of MSI/dMMR mutation-negative individuals and identification of two inactivating mutations in the tumor allow to exclude the patient and their relatives from heavy LS-specific surveillance protocols.

Moreover, in a recent study, the tumor sequencing of MMR genes among other genes, in addition to assessing the *BRAF* and MSI status in a series of patients with CRC, showed better sensitivity than MSI or IHC, followed by a *BRAF* mutation analysis (100% vs. 89.7%), with equal specificity (95.3% vs. 94.6%) for the identification of LS [297]. This result needs to be confirmed, as tumor sequencing is technically challenging (especially for CNV detection), and the cost-effectiveness has to be determined. However, this raises the question of introducing tumor sequencing first, with or without an *MLH1* methylation analysis, as a replacement for the current LS screening tests. The identification of one or more MMR mutations would lead to genetics counseling and further germline MMR analysis.

### 4.3. Optimal Strategy for Discrimination of LS-Related and Sporadic MSI/dMMR Tumors

**Figure 2:**
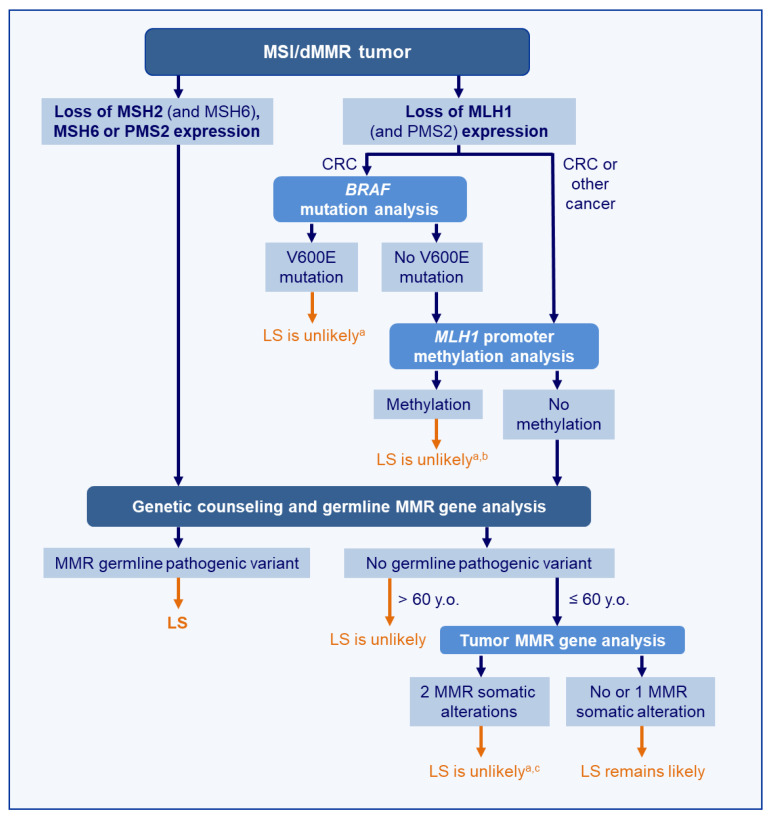
Molecular strategy to distinguish LS-related from sporadic MSI/dMMR tumors. ^a^ Should be interpreted with the patient’s clinical data and family history of cancer; thus, a strong suspicion of LS may lead to a further germline MMR analysis. ^b^ Although rare, the hypotheses of germline *MLH1* epimutation and of methylation as the 2nd hit should be considered and may lead to further germline *MLH1* analysis. ^c^ Although rare, the hypothesis of a mosaic MMR mutation should be considered. Abbreviation: LS, Lynch syndrome, CRC: Colorectal Cancer

A strategy for distinguishing LS and sporadic tumors is proposed in Figure 2. However, it is important to keep in mind that there is no marker allowing the perfect discrimination of LS-related and sporadic cancers in the context of screening. As indicated above, even the detection of *MLH1* promoter hypermethylation or of a *BRAF* mutation (in CRC) does not exclude definitively the possibility of LS, and it remains important to consider the results in light of the clinical data, i.e., the patient’s personal and family history of cancers. Moreover, although these markers are used to detect LS, they should not be considered as diagnostic markers of LS. Only the identification of a germline pathogenic variant (or epimutation) in one of the four MMR genes or *EPCAM* will confirm the diagnosis of LS and lead to appropriate management of the patient and his relatives.

The absence of germline mutation (or epimutation) detected in MMR genes (and in *MUTYH* or *POLE*) should lead to a further tumor analysis to look for biallelic somatic MMR gene mutations. This is of importance for appropriate genetic counseling and clinical management. Somatic MMR mutations are likely sporadic events. Therefore, the identification of two MMR mutations exclusively in the tumor (a mosaicism should have been excluded) will allow the reassurance of patients and their relatives and their exclusion from LS-specific surveillance programs. This led us to propose a tumoral MMR gene analysis in patients who developed their MSI/dMMR tumor before the age of 60 when no germline variant has been identified; as for patients above 60 y.o., a sporadic tumor is the most likely explanation and does not require further tumor testing [220].

## 5. Conclusions

The methods for LS diagnosis have considerably evolved in recent years and so have the tumoral tests for LS screening and for the discrimination of LS-related and sporadic MSI/dMMR tumors, leading to the improved identification of LS patients. In this review, we provided an update on the available clinical, histopathological, and molecular criteria that are evocative of LS. We also focused on the different mechanisms and pitfalls that can lead to potential misinterpretations and to inappropriate clinical management. A strategy taking into account these issues was proposed. Even if there are still a few cases with unknown etiology of the MSI/dMMR, the proposed screening strategy makes it possible to identify the vast majority of LS-related tumors and sporadic tumors, enabling the appropriate management of patients and their family.

## Figures and Tables

**Table 1:** Amsterdam II criteria [11] and revised Bethesda Guidelines [15].

**Amsterdam II Criteria**
(1) Three or more relatives with histologically verified LS-associated cancer (colorectal cancer, cancer of the endometrium, small bowel, ureter, or renal pelvis), 1 of which is a first-degree relative of the others. (2) Cancer involving at least 2 generations. (3) One or more cancer cases diagnosed before the age of 50. (4) Familial adenomatous polyposis should be excluded.
**Revised Bethesda Criteria ^1^**
(1) CRC or endometrial cancer diagnosed at age younger than 50. (2) Presence of synchronous or metachronous CRC or other LS-associated tumors, regardless of age. (3) CRC with MSI-high pathologic-associated features (Crohn-like lymphocytic reaction, mucinous/signet cell differentiation, or medullary growth pattern) diagnosed in an individual younger than 60. (4) Patient with CRC or LS-associated tumor diagnosed below 50 in at least 1 first-degree relative. (5) Patient with CRC or LS-associated tumor diagnosed at any age in 2 first-degree or second-degree relatives.

^1^ Only one of these criteria needs to be met. CRC: colorectal cancer, LS: Lynch syndrome, and MSI: microsatellite instability.

**Table 2:** Clinical and molecular features evocative of LS-related MSI/mismatch repair-deficient (dMMR) cancers.

Clinical Criteria/Molecular Test	Data Evocative of LS	Limitations
Clinical presentation	Personal and/or family history of (multiple) LS-related cancers [239] Age of cancer onset ≤ 60 y.o.: - 83% of CRC [16,17] - 65–89% of EC [20,43,44,45] - 90% of OC [60] - 78% of sebaceous tumors [92]	A number of LS patient develop their cancer > 60 y.o.: - 17% of CRC - 11–35% of EC - 10% of OC - 22% of sebaceous tumors
Histopathological characteristics	CRC: associated conventional adenomas; MMR-deficient crypts in peritumoral mucosa: Se 30–70%, Sp ≥ 99% [163,164,165] EC: increased number of CD8+ immune cells [51]	
IHC for MMR proteins	Loss of MSH2/MSH6, isolated loss of MSH6 or isolated loss of PMS2	Some may be due to somatic inactivation (up to 60% of sebaceous tumors with loss of MSH2/MSH6 [93,95]; up to 50% of isolated loss of PMS2 may be explained by *MLH1* hypermethylation [240])
*BRAF* mutation analysis (V600E)	Absence of BRAF V600E mutation CRC: Se 98.5%; Sp 66% [241]	BRAF mutation in 1.4% of CRC from LS patients [241] No utility in non-CRC
*MLH1* promoter methylation analysis	Absence of methylation CRC: Se 94%; Sp 88% [241]	Methylation in up to 6% of CRC from LS patients [242]

Abbreviations: EC, endometrial cancer; OC, ovarian cancer; Se, sensitivity; Sp, specificity; and IHC, immunohistochemistry.

## Data Availability

Not applicable.

## References

[1] Bonadona V., Bonaiti B., Olschwang S., Grandjouan S., Huiart L., Longy M., Guimbaud R., Buecher B., Bignon Y.J., Caron O. (2011). Cancer risks associated with germline mutations in MLH1, MSH2, and MSH6 genes in Lynch syndrome. JAMA.

[2] Moller P., Seppala T.T., Bernstein I., Holinski-Feder E., Sala P., Gareth Evans D., Lindblom A., Macrae F., Blanco I., Sijmons R.H. (2018). Cancer risk and survival in path_MMR carriers by gene and gender up to 75 years of age: A report from the Prospective Lynch Syndrome Database. Gut.

[3] Dominguez-Valentin M., Sampson J.R., Seppala T.T., Ten Broeke S.W., Plazzer J.P., Nakken S., Engel C., Aretz S., Jenkins M.A., Sunde L. (2020). Cancer risks by gene, age, and gender in 6350 carriers of pathogenic mismatch repair variants: Findings from the Prospective Lynch Syndrome Database. Genet. Med..

[4] Bucksch K., Zachariae S., Aretz S., Buttner R., Holinski-Feder E., Holzapfel S., Huneburg R., Kloor M., von Knebel Doeberitz M., Morak M. (2020). Cancer risks in Lynch syndrome, Lynch-like syndrome, and familial colorectal cancer type X: A prospective cohort study. BMC Cancer.

[5] Stoffel E.M., Mangu P.B., Gruber S.B., Hamilton S.R., Kalady M.F., Lau M.W., Lu K.H., Roach N., Limburg P.J. (2015). Hereditary colorectal cancer syndromes: American Society of Clinical Oncology Clinical Practice Guideline endorsement of the familial risk-colorectal cancer: European Society for Medical Oncology Clinical Practice Guidelines. J. Clin. Oncol..

[6] Vangala D.B., Cauchin E., Balmana J., Wyrwicz L., van Cutsem E., Guller U., Castells A., Carneiro F., Hammel P., Ducreux M. (2018). Screening and surveillance in hereditary gastrointestinal cancers: Recommendations from the European Society of Digestive Oncology (ESDO) expert discussion at the 20th European Society for Medical Oncology (ESMO)/World Congress on Gastrointestinal Cancer, Barcelona, June 2018. Eur. J. Cancer.

[7] Seppala T.T., Latchford A., Negoi I., Sampaio Soares A., Jimenez-Rodriguez R., Sanchez-Guillen L., Evans D.G., Ryan N., Crosbie E.J., Dominguez-Valentin M. (2020). European guidelines from the EHTG and ESCP for Lynch syndrome: An updated third edition of the Mallorca guidelines based on gene and gender. Br. J. Surg..

[8] Le D.T., Durham J.N., Smith K.N., Wang H., Bartlett B.R., Aulakh L.K., Lu S., Kemberling H., Wilt C., Luber B.S. (2017). Mismatch repair deficiency predicts response of solid tumors to PD-1 blockade. Science.

[9] Zaanan A., Shi Q., Taieb J., Alberts S.R., Meyers J.P., Smyrk T.C., Julie C., Zawadi A., Tabernero J., Mini E. (2020). Clinical Outcomes in Patients with Colon Cancer with Microsatellite Instability of Sporadic or Familial Origin Treated with Adjuvant FOLFOX With or without Cetuximab: A Pooled Analysis of the PETACC8 and N0147 Trials. JCO Precis. Oncol..

[10] Ten Broeke S.W., van der Klift H.M., Tops C.M.J., Aretz S., Bernstein I., Buchanan D.D., de la Chapelle A., Capella G., Clendenning M., Engel C. (2018). Cancer Risks for PMS2-Associated Lynch Syndrome. J. Clin. Oncol..

[11] Vasen H.F., Watson P., Mecklin J.P., Lynch H.T. (1999). New clinical criteria for hereditary nonpolyposis colorectal cancer (HNPCC, Lynch syndrome) proposed by the International Collaborative group on HNPCC. Gastroenterology.

[12] Barnetson R.A., Tenesa A., Farrington S.M., Nicholl I.D., Cetnarskyj R., Porteous M.E., Campbell H., Dunlop M.G. (2006). Identification and survival of carriers of mutations in DNA mismatch-repair genes in colon cancer. N. Engl. J. Med..

[13] Moreira L., Balaguer F., Lindor N., de la Chapelle A., Hampel H., Aaltonen L.A., Hopper J.L., Le Marchand L., Gallinger S., Newcomb P.A. (2012). Identification of Lynch syndrome among patients with colorectal cancer. JAMA.

[14] Giardiello F.M., Allen J.I., Axilbund J.E., Boland C.R., Burke C.A., Burt R.W., Church J.M., Dominitz J.A., Johnson D.A., Kaltenbach T. (2014). Guidelines on genetic evaluation and management of Lynch syndrome: A consensus statement by the US Multi-Society Task Force on colorectal cancer. Gastroenterology.

[15] Umar A., Boland C.R., Terdiman J.P., Syngal S., de la Chapelle A., Ruschoff J., Fishel R., Lindor N.M., Burgart L.J., Hamelin R. (2004). Revised Bethesda Guidelines for hereditary nonpolyposis colorectal cancer (Lynch syndrome) and microsatellite instability. J. Natl. Cancer Inst..

[16] Hampel H., Frankel W.L., Martin E., Arnold M., Khanduja K., Kuebler P., Nakagawa H., Sotamaa K., Prior T.W., Westman J. (2005). Screening for the Lynch syndrome (hereditary nonpolyposis colorectal cancer). N. Engl. J. Med..

[17] Hampel H., Frankel W.L., Martin E., Arnold M., Khanduja K., Kuebler P., Clendenning M., Sotamaa K., Prior T., Westman J.A. (2008). Feasibility of screening for Lynch syndrome among patients with colorectal cancer. J. Clin. Oncol..

[18] Kahn R.M., Gordhandas S., Maddy B.P., Baltich Nelson B., Askin G., Christos P.J., Caputo T.A., Chapman-Davis E., Holcomb K., Frey M.K. (2019). Universal endometrial cancer tumor typing: How much has immunohistochemistry, microsatellite instability, and MLH1 methylation improved the diagnosis of Lynch syndrome across the population?. Cancer.

[19] Mills A.M., Liou S., Ford J.M., Berek J.S., Pai R.K., Longacre T.A. (2014). Lynch syndrome screening should be considered for all patients with newly diagnosed endometrial cancer. Am. J. Surg. Pathol..

[20] Goodfellow P.J., Billingsley C.C., Lankes H.A., Ali S., Cohn D.E., Broaddus R.J., Ramirez N., Pritchard C.C., Hampel H., Chassen A.S. (2015). Combined Microsatellite Instability, MLH1 Methylation Analysis, and Immunohistochemistry for Lynch Syndrome Screening in Endometrial Cancers From GOG210: An NRG Oncology and Gynecologic Oncology Group Study. J. Clin. Oncol..

[21] Palomaki G.E., McClain M.R., Melillo S., Hampel H.L., Thibodeau S.N. (2009). EGAPP supplementary evidence review: DNA testing strategies aimed at reducing morbidity and mortality from Lynch syndrome. Genet. Med..

[22] Provenzale D., Gupta S., Ahnen D.J., Bray T., Cannon J.A., Cooper G., David D.S., Early D.S., Erwin D., Ford J.M. (2016). Genetic/Familial High-Risk Assessment: Colorectal Version 1.2016, NCCN Clinical Practice Guidelines in Oncology. J. Natl. Compr. Cancer Netw..

[23] Syngal S., Brand R.E., Church J.M., Giardiello F.M., Hampel H.L., Burt R.W. (2015). ACG clinical guideline: Genetic testing and management of hereditary gastrointestinal cancer syndromes. Am. J. Gastroenterol..

[24] Vasen H.F., Blanco I., Aktan-Collan K., Gopie J.P., Alonso A., Aretz S., Bernstein I., Bertario L., Burn J., Capella G. (2013). Revised guidelines for the clinical management of Lynch syndrome (HNPCC): Recommendations by a group of European experts. Gut.

[25] Ju J.Y., Mills A.M., Mahadevan M.S., Fan J., Culp S.H., Thomas M.H., Cathro H.P. (2018). Universal Lynch Syndrome Screening Should be Performed in All Upper Tract Urothelial Carcinomas. Am. J. Surg. Pathol..

[26] Orta L., Klimstra D.S., Qin J., Mecca P., Tang L.H., Busam K.J., Shia J. (2009). Towards identification of hereditary DNA mismatch repair deficiency: Sebaceous neoplasm warrants routine immunohistochemical screening regardless of patient’s age or other clinical characteristics. Am. J. Surg. Pathol..

[27] Young J., Simms L.A., Biden K.G., Wynter C., Whitehall V., Karamatic R., George J., Goldblatt J., Walpole I., Robin S.A. (2001). Features of colorectal cancers with high-level microsatellite instability occurring in familial and sporadic settings: Parallel pathways of tumorigenesis. Am. J. Pathol..

[28] Halvarsson B., Anderson H., Domanska K., Lindmark G., Nilbert M. (2008). Clinicopathologic factors identify sporadic mismatch repair-defective colon cancers. Am. J. Clin. Pathol..

[29] Hartman D.J., Brand R.E., Hu H., Bahary N., Dudley B., Chiosea S.I., Nikiforova M.N., Pai R.K. (2013). Lynch syndrome-associated colorectal carcinoma: Frequent involvement of the left colon and rectum and late-onset presentation supports a universal screening approach. Hum. Pathol..

[30] Yamada R., Yamaguchi T., Iijima T., Wakaume R., Takao M., Koizumi K., Hishima T., Horiguchi S.I. (2018). Differences in histological features and PD-L1 expression between sporadic microsatellite instability and Lynch-syndrome-associated disease in Japanese patients with colorectal cancer. Int. J. Clin. Oncol..

[31] Moller P., Seppala T., Bernstein I., Holinski-Feder E., Sala P., Evans D.G., Lindblom A., Macrae F., Blanco I., Sijmons R. (2017). Cancer incidence and survival in Lynch syndrome patients receiving colonoscopic and gynaecological surveillance: First report from the prospective Lynch syndrome database. Gut.

[32] Mas-Moya J., Dudley B., Brand R.E., Thull D., Bahary N., Nikiforova M.N., Pai R.K. (2015). Clinicopathological comparison of colorectal and endometrial carcinomas in patients with Lynch-like syndrome versus patients with Lynch syndrome. Hum. Pathol..

[33] Jass J.R. (2007). Classification of colorectal cancer based on correlation of clinical, morphological and molecular features. Histopathology.

[34] Shia J., Ellis N.A., Paty P.B., Nash G.M., Qin J., Offit K., Zhang X.M., Markowitz A.J., Nafa K., Guillem J.G. (2003). Value of histopathology in predicting microsatellite instability in hereditary nonpolyposis colorectal cancer and sporadic colorectal cancer. Am. J. Surg. Pathol..

[35] Gologan A., Sepulveda A.R. (2005). Microsatellite instability and DNA mismatch repair deficiency testing in hereditary and sporadic gastrointestinal cancers. Clin. Lab. Med..

[36] Jenkins M.A., Hayashi S., O’Shea A.M., Burgart L.J., Smyrk T.C., Shimizu D., Waring P.M., Ruszkiewicz A.R., Pollett A.F., Redston M. (2007). Pathology features in Bethesda guidelines predict colorectal cancer microsatellite instability: A population-based study. Gastroenterology.

[37] Rosenbaum M.W., Bledsoe J.R., Morales-Oyarvide V., Huynh T.G., Mino-Kenudson M. (2016). PD-L1 expression in colorectal cancer is associated with microsatellite instability, BRAF mutation, medullary morphology and cytotoxic tumor-infiltrating lymphocytes. Mod. Pathol..

[38] Yearsley M., Hampel H., Lehman A., Nakagawa H., de la Chapelle A., Frankel W.L. (2006). Histologic features distinguish microsatellite-high from microsatellite-low and microsatellite-stable colorectal carcinomas, but do not differentiate germline mutations from methylation of the MLH1 promoter. Hum. Pathol..

[39] Hemminger J.A., Pearlman R., Haraldsdottir S., Knight D., Jonasson J.G., Pritchard C.C., Hampel H., Frankel W.L. (2018). Histology of colorectal adenocarcinoma with double somatic mismatch-repair mutations is indistinguishable from those caused by Lynch syndrome. Hum. Pathol..

[40] Cosgrove C.M., Cohn D.E., Hampel H., Frankel W.L., Jones D., McElroy J.P., Suarez A.A., Zhao W., Chen W., Salani R. (2017). Epigenetic silencing of MLH1 in endometrial cancers is associated with larger tumor volume, increased rate of lymph node positivity and reduced recurrence-free survival. Gynecol. Oncol..

[41] Rossi L., Le Frere-Belda M.A., Laurent-Puig P., Buecher B., De Pauw A., Stoppa-Lyonnet D., Canlorbe G., Caron O., Borghese B., Colas C. (2017). Clinicopathologic Characteristics of Endometrial Cancer in Lynch Syndrome: A French Multicenter Study. Int. J. Gynecol. Cancer.

[42] Broaddus R.R., Lynch H.T., Chen L.M., Daniels M.S., Conrad P., Munsell M.F., White K.G., Luthra R., Lu K.H. (2006). Pathologic features of endometrial carcinoma associated with HNPCC: A comparison with sporadic endometrial carcinoma. Cancer.

[43] Hampel H., Frankel W., Panescu J., Lockman J., Sotamaa K., Fix D., Comeras I., La Jeunesse J., Nakagawa H., Westman J.A. (2006). Screening for Lynch syndrome (hereditary nonpolyposis colorectal cancer) among endometrial cancer patients. Cancer Res..

[44] Leenen C.H., van Lier M.G., van Doorn H.C., van Leerdam M.E., Kooi S.G., de Waard J., Hoedemaeker R.F., van den Ouweland A.M., Hulspas S.M., Dubbink H.J. (2012). Prospective evaluation of molecular screening for Lynch syndrome in patients with endometrial cancer </= 70 years. Gynecol. Oncol..

[45] Buchanan D.D., Tan Y.Y., Walsh M.D., Clendenning M., Metcalf A.M., Ferguson K., Arnold S.T., Thompson B.A., Lose F.A., Parsons M.T. (2014). Tumor mismatch repair immunohistochemistry and DNA MLH1 methylation testing of patients with endometrial cancer diagnosed at age younger than 60 years optimizes triage for population-level germline mismatch repair gene mutation testing. J. Clin. Oncol..

[46] Bruegl A.S., Djordjevic B., Urbauer D.L., Westin S.N., Soliman P.T., Lu K.H., Luthra R., Broaddus R.R. (2014). Utility of MLH1 methylation analysis in the clinical evaluation of Lynch Syndrome in women with endometrial cancer. Curr. Pharm. Des..

[47] Shia J., Black D., Hummer A.J., Boyd J., Soslow R.A. (2008). Routinely assessed morphological features correlate with microsatellite instability status in endometrial cancer. Hum. Pathol..

[48] Garg K., Soslow R.A. (2009). Lynch syndrome (hereditary non-polyposis colorectal cancer) and endometrial carcinoma. J. Clin. Pathol..

[49] Honore L.H., Hanson J., Andrew S.E. (2006). Microsatellite instability in endometrioid endometrial carcinoma: Correlation with clinically relevant pathologic variables. Int. J. Gynecol. Cancer.

[50] Walsh M.D., Cummings M.C., Buchanan D.D., Dambacher W.M., Arnold S., McKeone D., Byrnes R., Barker M.A., Leggett B.A., Gattas M. (2008). Molecular, pathologic, and clinical features of early-onset endometrial cancer: Identifying presumptive Lynch syndrome patients. Clin. Cancer Res..

[51] Ramchander N.C., Ryan N.A.J., Walker T.D.J., Harries L., Bolton J., Bosse T., Evans D.G., Crosbie E.J. (2019). Distinct Immunological Landscapes Characterize Inherited and Sporadic Mismatch Repair Deficient Endometrial Cancer. Front. Immunol..

[52] Djordjevic B., Barkoh B.A., Luthra R., Broaddus R.R. (2013). Relationship between PTEN, DNA mismatch repair, and tumor histotype in endometrial carcinoma: Retained positive expression of PTEN preferentially identifies sporadic non-endometrioid carcinomas. Mod. Pathol..

[53] Sloan E.A., Moskaluk C.A., Mills A.M. (2017). Mucinous Differentiation with Tumor Infiltrating Lymphocytes Is a Feature of Sporadically Methylated Endometrial Carcinomas. Int. J. Gynecol. Pathol..

[54] (2014). ACOG Practice Bulletin No. 147: Lynch syndrome. Obs. Gynecol..

[55] Lancaster J.M., Powell C.B., Chen L.M., Richardson D.L. (2015). Society of Gynecologic Oncology statement on risk assessment for inherited gynecologic cancer predispositions. Gynecol. Oncol..

[56] Leskela S., Romero I., Cristobal E., Perez-Mies B., Rosa-Rosa J.M., Gutierrez-Pecharroman A., Caniego-Casas T., Santon A., Ojeda B., Lopez-Reig R. (2020). Mismatch Repair Deficiency in Ovarian Carcinoma: Frequency, Causes, and Consequences. Am. J. Surg. Pathol..

[57] Aysal A., Karnezis A., Medhi I., Grenert J.P., Zaloudek C.J., Rabban J.T. (2012). Ovarian endometrioid adenocarcinoma: Incidence and clinical significance of the morphologic and immunohistochemical markers of mismatch repair protein defects and tumor microsatellite instability. Am. J. Surg. Pathol..

[58] Xiao X., Dong D., He W., Song L., Wang Q., Yue J., Xie L. (2018). Mismatch repair deficiency is associated with MSI phenotype, increased tumor-infiltrating lymphocytes and PD-L1 expression in immune cells in ovarian cancer. Gynecol. Oncol..

[59] Watson P., Butzow R., Lynch H.T., Mecklin J.P., Jarvinen H.J., Vasen H.F., Madlensky L., Fidalgo P., Bernstein I. (2001). The clinical features of ovarian cancer in hereditary nonpolyposis colorectal cancer. Gynecol. Oncol..

[60] Ketabi Z., Bartuma K., Bernstein I., Malander S., Gronberg H., Bjorck E., Holck S., Nilbert M. (2011). Ovarian cancer linked to Lynch syndrome typically presents as early-onset, non-serous epithelial tumors. Gynecol. Oncol..

[61] Grindedal E.M., Renkonen-Sinisalo L., Vasen H., Evans G., Sala P., Blanco I., Gronwald J., Apold J., Eccles D.M., Sanchez A.A. (2010). Survival in women with MMR mutations and ovarian cancer: A multicentre study in Lynch syndrome kindreds. J. Med. Genet..

[62] Niskakoski A., Kaur S., Renkonen-Sinisalo L., Lassus H., Jarvinen H.J., Mecklin J.P., Butzow R., Peltomaki P. (2013). Distinct molecular profiles in Lynch syndrome-associated and sporadic ovarian carcinomas. Int. J. Cancer.

[63] Helder-Woolderink J.M., Blok E.A., Vasen H.F., Hollema H., Mourits M.J., De Bock G.H. (2016). Ovarian cancer in Lynch syndrome; a systematic review. Eur. J. Cancer.

[64] Woolderink J.M., De Bock G.H., de Hullu J.A., Hollema H., Zweemer R.P., Slangen B.F.M., Gaarenstroom K.N., van Beurden M., van Doorn H.C., Sijmons R.H. (2018). Characteristics of Lynch syndrome associated ovarian cancer. Gynecol. Oncol..

[65] Urakami S., Inoshita N., Oka S., Miyama Y., Nomura S., Arai M., Sakaguchi K., Kurosawa K., Okaneya T. (2018). Clinicopathological characteristics of patients with upper urinary tract urothelial cancer with loss of immunohistochemical expression of the DNA mismatch repair proteins in universal screening. Int. J. Urol..

[66] Harper H.L., McKenney J.K., Heald B., Stephenson A., Campbell S.C., Plesec T., Magi-Galluzzi C. (2017). Upper tract urothelial carcinomas: Frequency of association with mismatch repair protein loss and lynch syndrome. Mod. Pathol..

[67] Gayhart M.G., Johnson N., Paul A., Quillin J.M., Hampton L.J., Idowu M.O., Smith S.C. (2020). Universal Mismatch Repair Protein Screening in Upper Tract Urothelial Carcinoma. Am. J. Clin. Pathol..

[68] Metcalfe M.J., Petros F.G., Rao P., Mork M.E., Xiao L., Broaddus R.R., Matin S.F. (2018). Universal Point of Care Testing for Lynch Syndrome in Patients with Upper Tract Urothelial Carcinoma. J. Urol..

[69] Gylling A.H., Nieminen T.T., Abdel-Rahman W.M., Nuorva K., Juhola M., Joensuu E.I., Jarvinen H.J., Mecklin J.P., Aarnio M., Peltomaki P.T. (2008). Differential cancer predisposition in Lynch syndrome: Insights from molecular analysis of brain and urinary tract tumors. Carcinogenesis.

[70] Crockett D.G., Wagner D.G., Holmang S., Johansson S.L., Lynch H.T. (2011). Upper urinary tract carcinoma in Lynch syndrome cases. J. Urol..

[71] Roupret M., Yates D.R., Comperat E., Cussenot O. (2008). Upper urinary tract urothelial cell carcinomas and other urological malignancies involved in the hereditary nonpolyposis colorectal cancer (lynch syndrome) tumor spectrum. Eur. Urol..

[72] Hartmann A., Dietmaier W., Hofstadter F., Burgart L.J., Cheville J.C., Blaszyk H. (2003). Urothelial carcinoma of the upper urinary tract: Inverted growth pattern is predictive of microsatellite instability. Hum. Pathol..

[73] Joost P., Therkildsen C., Dominguez-Valentin M., Jonsson M., Nilbert M. (2015). Urinary Tract Cancer in Lynch Syndrome; Increased Risk in Carriers of MSH2 Mutations. Urology.

[74] Kim H., An J.Y., Noh S.H., Shin S.K., Lee Y.C. (2011). High microsatellite instability predicts good prognosis in intestinal-type gastric cancers. J. Gastroenterol. Hepatol..

[75] Polom K., Marano L., Marrelli D., De Luca R., Roviello G., Savelli V., Tan P., Roviello F. (2018). Meta-analysis of microsatellite instability in relation to clinicopathological characteristics and overall survival in gastric cancer. Br. J. Surg..

[76] Kim K.J., Lee T.H., Cho N.Y., Yang H.K., Kim W.H., Kang G.H. (2013). Differential clinicopathologic features in microsatellite-unstable gastric cancers with and without MLH1 methylation. Hum. Pathol..

[77] Fornasarig M., Magris R., De Re V., Bidoli E., Canzonieri V., Maiero S., Viel A., Cannizzaro R. (2018). Molecular and Pathological Features of Gastric Cancer in Lynch Syndrome and Familial Adenomatous Polyposis. Int. J. Mol. Sci..

[78] Kim J., Braun D., Ukaegbu C., Dhingra T.G., Kastrinos F., Parmigiani G., Syngal S., Yurgelun M.B. (2020). Clinical Factors Associated With Gastric Cancer in Individuals With Lynch Syndrome. Clin. Gastroenterol. Hepatol..

[79] Ju J.Y., Dibbern M.E., Mahadevan M.S., Fan J., Kunk P.R., Stelow E.B. (2020). Mismatch Repair Protein Deficiency/Microsatellite Instability Is Rare in Cholangiocarcinomas and Associated With Distinctive Morphologies. Am. J. Clin. Pathol..

[80] Silva V.W., Askan G., Daniel T.D., Lowery M., Klimstra D.S., Abou-Alfa G.K., Shia J. (2016). Biliary carcinomas: Pathology and the role of DNA mismatch repair deficiency. Chin. Clin. Oncol..

[81] Aparicio T., Svrcek M., Zaanan A., Beohou E., Laforest A., Afchain P., Mitry E., Taieb J., Di Fiore F., Gornet J.M. (2013). Small bowel adenocarcinoma phenotyping, a clinicobiological prognostic study. Br. J. Cancer.

[82] Ten Kate G.L., Kleibeuker J.H., Nagengast F.M., Craanen M., Cats A., Menko F.H., Vasen H.F. (2007). Is surveillance of the small bowel indicated for Lynch syndrome families?. Gut.

[83] Lupinacci R.M., Goloudina A., Buhard O., Bachet J.B., Marechal R., Demetter P., Cros J., Bardier-Dupas A., Collura A., Cervera P. (2018). Prevalence of Microsatellite Instability in Intraductal Papillary Mucinous Neoplasms of the Pancreas. Gastroenterology.

[84] Grover S., Syngal S. (2010). Hereditary pancreatic cancer. Gastroenterology.

[85] Banville N., Geraghty R., Fox E., Leahy D.T., Green A., Keegan D., Geoghegan J., O’Donoghue D., Hyland J., Sheahan K. (2006). Medullary carcinoma of the pancreas in a man with hereditary nonpolyposis colorectal cancer due to a mutation of the MSH2 mismatch repair gene. Hum. Pathol..

[86] Bujanda L., Herreros-Villanueva M. (2017). Pancreatic Cancer in Lynch Syndrome Patients. J. Cancer.

[87] Flanagan M.R., Jayaraj A., Xiong W., Yeh M.M., Raskind W.H., Pillarisetty V.G. (2015). Pancreatic intraductal papillary mucinous neoplasm in a patient with Lynch syndrome. World J. Gastroenterol..

[88] Sparr J.A., Bandipalliam P., Redston M.S., Syngal S. (2009). Intraductal papillary mucinous neoplasm of the pancreas with loss of mismatch repair in a patient with Lynch syndrome. Am. J. Surg. Pathol..

[89] Lee S.H., Kim W.Y., Hwang D.Y., Han H.S. (2015). Intraductal papillary mucinous neoplasm of the ileal heterotopic pancreas in a patient with hereditary non-polyposis colorectal cancer: A case report. World J. Gastroenterol..

[90] Bhaijee F., Brown A.S. (2014). Muir-Torre syndrome. Arch. Pathol. Lab. Med..

[91] Ferreira I., Wiedemeyer K., Demetter P., Adams D.J., Arends M.J., Brenn T. (2020). Update on the pathology, genetics and somatic landscape of sebaceous tumours. Histopathology.

[92] Everett J.N., Raymond V.M., Dandapani M., Marvin M., Kohlmann W., Chittenden A., Koeppe E., Gustafson S.L., Else T., Fullen D.R. (2014). Screening for germline mismatch repair mutations following diagnosis of sebaceous neoplasm. JAMA Derm..

[93] Plocharczyk E.F., Frankel W.L., Hampel H., Peters S.B. (2013). Mismatch repair protein deficiency is common in sebaceous neoplasms and suggests the importance of screening for Lynch syndrome. Am. J. Derm..

[94] Roberts M.E., Riegert-Johnson D.L., Thomas B.C., Thomas C.S., Heckman M.G., Krishna M., DiCaudo D.J., Bridges A.G., Hunt K.S., Rumilla K.M. (2013). Screening for Muir-Torre syndrome using mismatch repair protein immunohistochemistry of sebaceous neoplasms. J. Genet. Couns.

[95] Kruse R., Rutten A., Schweiger N., Jakob E., Mathiak M., Propping P., Mangold E., Bisceglia M., Ruzicka T. (2003). Frequency of microsatellite instability in unselected sebaceous gland neoplasias and hyperplasias. J. Investig. Derm..

[96] Navi D., Wadhera A., Fung M.A., Fazel N. (2006). Muir-Torre syndrome. Derm. Online J..

[97] South C.D., Hampel H., Comeras I., Westman J.A., Frankel W.L., de la Chapelle A. (2008). The frequency of Muir-Torre syndrome among Lynch syndrome families. J. Natl. Cancer Inst..

[98] Ponti G., Losi L., Di Gregorio C., Roncucci L., Pedroni M., Scarselli A., Benatti P., Seidenari S., Pellacani G., Lembo L. (2005). Identification of Muir-Torre syndrome among patients with sebaceous tumors and keratoacanthomas: Role of clinical features, microsatellite instability, and immunohistochemistry. Cancer.

[99] Akhtar S., Oza K.K., Khan S.A., Wright J. (1999). Muir-Torre syndrome: Case report of a patient with concurrent jejunal and ureteral cancer and a review of the literature. J. Am. Acad. Derm..

[100] Walsh M.D., Jayasekara H., Huang A., Winship I.M., Buchanan D.D. (2019). Clinico-pathological predictors of mismatch repair deficiency in sebaceous neoplasia: A large case series from a single Australian private pathology service. Australas J. Derm..

[101] Stewart W.M., Lauret P., Hemet J., Thomine E., Gueville R.M. (1977). Multiple kerato-acanthomas and visceral carcinomas: Torre’s syndrome. Ann. Derm. Venereol.

[102] Latham A., Srinivasan P., Kemel Y., Shia J., Bandlamudi C., Mandelker D., Middha S., Hechtman J., Zehir A., Dubard-Gault M. (2019). Microsatellite Instability Is Associated With the Presence of Lynch Syndrome Pan-Cancer. J. Clin. Oncol..

[103] Campbell B.B., Light N., Fabrizio D., Zatzman M., Fuligni F., de Borja R., Davidson S., Edwards M., Elvin J.A., Hodel K.P. (2017). Comprehensive Analysis of Hypermutation in Human Cancer. Cell.

[104] Shlien A., Campbell B.B., de Borja R., Alexandrov L.B., Merico D., Wedge D., Van Loo P., Tarpey P.S., Coupland P., Behjati S. (2015). Combined hereditary and somatic mutations of replication error repair genes result in rapid onset of ultra-hypermutated cancers. Nat. Genet..

[105] Yang C., Austin F., Richard H., Idowu M., Williamson V., Sabato F., Ferreira-Gonzalez A., Turner S.A. (2019). Lynch syndrome-associated ultra-hypermutated pediatric glioblastoma mimicking a constitutional mismatch repair deficiency syndrome. Cold Spring Harb. Mol. Case Stud..

[106] Barresi V., Simbolo M., Mafficini A., Piredda M.L., Caffo M., Cardali S.M., Germano A., Cingarlini S., Ghimenton C., Scarpa A. (2019). Ultra-Mutation in IDH Wild-Type Glioblastomas of Patients Younger than 55 Years is Associated with Defective Mismatch Repair, Microsatellite Instability, and Giant Cell Enrichment. Cancers.

[107] Therkildsen C., Ladelund S., Rambech E., Persson A., Petersen A., Nilbert M. (2015). Glioblastomas, astrocytomas and oligodendrogliomas linked to Lynch syndrome. Eur. J. Neurol..

[108] Erson-Omay E.Z., Caglayan A.O., Schultz N., Weinhold N., Omay S.B., Ozduman K., Koksal Y., Li J., Serin Harmanci A., Clark V. (2015). Somatic POLE mutations cause an ultramutated giant cell high-grade glioma subtype with better prognosis. Neuro Oncol..

[109] Shi Z.F., Li K.K., Kwan J.S.H., Yang R.R., Aibaidula A., Tang Q., Bao Y., Mao Y., Chen H., Ng H.K. (2019). Whole-exome sequencing revealed mutational profiles of giant cell glioblastomas. Brain Pathol..

[110] Boland C.R., Thibodeau S.N., Hamilton S.R., Sidransky D., Eshleman J.R., Burt R.W., Meltzer S.J., Rodriguez-Bigas M.A., Fodde R., Ranzani G.N. (1998). A National Cancer Institute Workshop on Microsatellite Instability for cancer detection and familial predisposition: Development of international criteria for the determination of microsatellite instability in colorectal cancer. Cancer Res..

[111] Buhard O., Suraweera N., Lectard A., Duval A., Hamelin R. (2004). Quasimonomorphic mononucleotide repeats for high-level microsatellite instability analysis. Dis. Markers.

[112] Suraweera N., Duval A., Reperant M., Vaury C., Furlan D., Leroy K., Seruca R., Iacopetta B., Hamelin R. (2002). Evaluation of tumor microsatellite instability using five quasimonomorphic mononucleotide repeats and pentaplex PCR. Gastroenterology.

[113] Xicola R.M., Llor X., Pons E., Castells A., Alenda C., Pinol V., Andreu M., Castellvi-Bel S., Paya A., Jover R. (2007). Performance of different microsatellite marker panels for detection of mismatch repair-deficient colorectal tumors. J. Natl. Cancer Inst..

[114] Goel A., Nagasaka T., Hamelin R., Boland C.R. (2010). An optimized pentaplex PCR for detecting DNA mismatch repair-deficient colorectal cancers. PLoS ONE.

[115] Luchini C., Bibeau F., Ligtenberg M.J.L., Singh N., Nottegar A., Bosse T., Miller R., Riaz N., Douillard J.Y., Andre F. (2019). ESMO recommendations on microsatellite instability testing for immunotherapy in cancer, and its relationship with PD-1/PD-L1 expression and tumour mutational burden: A systematic review-based approach. Ann. Oncol..

[116] Ferreira A.M., Westers H., Sousa S., Wu Y., Niessen R.C., Olderode-Berends M., van der Sluis T., Reuvekamp P.T., Seruca R., Kleibeuker J.H. (2009). Mononucleotide precedes dinucleotide repeat instability during colorectal tumour development in Lynch syndrome patients. J. Pathol..

[117] You J.F., Buhard O., Ligtenberg M.J., Kets C.M., Niessen R.C., Hofstra R.M., Wagner A., Dinjens W.N., Colas C., Lascols O. (2010). Tumours with loss of MSH6 expression are MSI-H when screened with a pentaplex of five mononucleotide repeats. Br. J. Cancer.

[118] Pagin A., Zerimech F., Leclerc J., Wacrenier A., Lejeune S., Descarpentries C., Escande F., Porchet N., Buisine M.P. (2013). Evaluation of a new panel of six mononucleotide repeat markers for the detection of DNA mismatch repair-deficient tumours. Br. J. Cancer.

[119] Hendriks Y.M., Wagner A., Morreau H., Menko F., Stormorken A., Quehenberger F., Sandkuijl L., Moller P., Genuardi M., Van Houwelingen H. (2004). Cancer risk in hereditary nonpolyposis colorectal cancer due to MSH6 mutations: Impact on counseling and surveillance. Gastroenterology.

[120] Wang Q., Leclerc J., Bougeard G., Olschwang S., Vasseur S., Cassinari K., Boidin D., Lefol C., Naibo P., Frebourg T. (2020). Characterisation of heterozygous PMS2 variants in French patients with Lynch syndrome. J. Med. Genet..

[121] Kuismanen S.A., Moisio A.L., Schweizer P., Truninger K., Salovaara R., Arola J., Butzow R., Jiricny J., Nystrom-Lahti M., Peltomaki P. (2002). Endometrial and colorectal tumors from patients with hereditary nonpolyposis colon cancer display different patterns of microsatellite instability. Am. J. Pathol..

[122] Wong Y.F., Cheung T.H., Lo K.W., Yim S.F., Chan L.K., Buhard O., Duval A., Chung T.K., Hamelin R. (2006). Detection of microsatellite instability in endometrial cancer: Advantages of a panel of five mononucleotide repeats over the National Cancer Institute panel of markers. Carcinogenesis.

[123] Libera L., Sahnane N., Carnevali I.W., Cimetti L., Cerutti R., Chiaravalli A.M., Riva C., Tibiletti M.G., Sessa F., Furlan D. (2017). Microsatellite analysis of sporadic and hereditary gynaecological cancer in routine diagnostics. J. Clin. Pathol..

[124] Mongiat-Artus P., Miquel C., Van der Aa M., Buhard O., Hamelin R., Soliman H., Bangma C., Janin A., Teillac P., van der Kwast T. (2006). Microsatellite instability and mutation analysis of candidate genes in urothelial cell carcinomas of upper urinary tract. Oncogene.

[125] Wang Y., Shi C., Eisenberg R., Vnencak-Jones C.L. (2017). Differences in Microsatellite Instability Profiles between Endometrioid and Colorectal Cancers: A Potential Cause for False-Negative Results?. J. Mol. Diagn..

[126] Bakry D., Aronson M., Durno C., Rimawi H., Farah R., Alharbi Q.K., Alharbi M., Shamvil A., Ben-Shachar S., Mistry M. (2014). Genetic and clinical determinants of constitutional mismatch repair deficiency syndrome: Report from the constitutional mismatch repair deficiency consortium. Eur. J. Cancer.

[127] Lavoine N., Colas C., Muleris M., Bodo S., Duval A., Entz-Werle N., Coulet F., Cabaret O., Andreiuolo F., Charpy C. (2015). Constitutional mismatch repair deficiency syndrome: Clinical description in a French cohort. J. Med. Genet..

[128] Touat M., Li Y.Y., Boynton A.N., Spurr L.F., Iorgulescu J.B., Bohrson C.L., Cortes-Ciriano I., Birzu C., Geduldig J.E., Pelton K. (2020). Mechanisms and therapeutic implications of hypermutation in gliomas. Nature.

[129] Pawlik T.M., Raut C.P., Rodriguez-Bigas M.A. (2004). Colorectal carcinogenesis: MSI-H versus MSI-L. Dis. Markers.

[130] Poynter J.N., Siegmund K.D., Weisenberger D.J., Long T.I., Thibodeau S.N., Lindor N., Young J., Jenkins M.A., Hopper J.L., Baron J.A. (2008). Molecular characterization of MSI-H colorectal cancer by MLHI promoter methylation, immunohistochemistry, and mismatch repair germline mutation screening. Cancer Epidemiol. Biomark. Prev..

[131] Coelho H., Jones-Hughes T., Snowsill T., Briscoe S., Huxley N., Frayling I.M., Hyde C. (2017). A systematic review of test accuracy studies evaluating molecular micro-satellite instability testing for the detection of individuals with lynch syndrome. BMC Cancer.

[132] Stelloo E., Jansen A.M.L., Osse E.M., Nout R.A., Creutzberg C.L., Ruano D., Church D.N., Morreau H., Smit V., van Wezel T. (2017). Practical guidance for mismatch repair-deficiency testing in endometrial cancer. Ann. Oncol..

[133] Snowsill T., Coelho H., Huxley N., Jones-Hughes T., Briscoe S., Frayling I.M., Hyde C. (2017). Molecular testing for Lynch syndrome in people with colorectal cancer: Systematic reviews and economic evaluation. Health Technol. Assess..

[134] Papke D.J., Nowak J.A., Yurgelun M.B., Frieden A., Srivastava A., Lindeman N.I., Sholl L.M., MacConaill L.E., Dong F. (2018). Validation of a targeted next-generation sequencing approach to detect mismatch repair deficiency in colorectal adenocarcinoma. Mod. Pathol..

[135] Yamamoto H., Imai K. (2019). An updated review of microsatellite instability in the era of next-generation sequencing and precision medicine. Semin. Oncol..

[136] Kautto E.A., Bonneville R., Miya J., Yu L., Krook M.A., Reeser J.W., Roychowdhury S. (2017). Performance evaluation for rapid detection of pan-cancer microsatellite instability with MANTIS. Oncotarget.

[137] Mojtahed A., Schrijver I., Ford J.M., Longacre T.A., Pai R.K. (2011). A two-antibody mismatch repair protein immunohistochemistry screening approach for colorectal carcinomas, skin sebaceous tumors, and gynecologic tract carcinomas. Mod. Pathol..

[138] O’Regan T., Chau K., Tatton M., Smith T., Parry S., Bissett I. (2013). Immunochemistry screening for Lynch syndrome in colorectal adenocarcinoma using an initial two antibody panel can replace a four antibody panel. N. Z. Med. J..

[139] Pearlman R., Markow M., Knight D., Chen W., Arnold C.A., Pritchard C.C., Hampel H., Frankel W.L. (2018). Two-stain immunohistochemical screening for Lynch syndrome in colorectal cancer may fail to detect mismatch repair deficiency. Mod. Pathol..

[140] Shia J., Klimstra D.S., Nafa K., Offit K., Guillem J.G., Markowitz A.J., Gerald W.L., Ellis N.A. (2005). Value of immunohistochemical detection of DNA mismatch repair proteins in predicting germline mutation in hereditary colorectal neoplasms. Am. J. Surg. Pathol..

[141] Southey M.C., Jenkins M.A., Mead L., Whitty J., Trivett M., Tesoriero A.A., Smith L.D., Jennings K., Grubb G., Royce S.G. (2005). Use of molecular tumor characteristics to prioritize mismatch repair gene testing in early-onset colorectal cancer. J. Clin. Oncol..

[142] Salahshor S., Koelble K., Rubio C., Lindblom A. (2001). Microsatellite Instability and hMLH1 and hMSH2 expression analysis in familial and sporadic colorectal cancer. Lab. Investig..

[143] Mangold E., Pagenstecher C., Friedl W., Fischer H.P., Merkelbach-Bruse S., Ohlendorf M., Friedrichs N., Aretz S., Buettner R., Propping P. (2005). Tumours from MSH2 mutation carriers show loss of MSH2 expression but many tumours from MLH1 mutation carriers exhibit weak positive MLH1 staining. J. Pathol..

[144] Wahlberg S.S., Schmeits J., Thomas G., Loda M., Garber J., Syngal S., Kolodner R.D., Fox E. (2002). Evaluation of microsatellite instability and immunohistochemistry for the prediction of germ-line MSH2 and MLH1 mutations in hereditary nonpolyposis colon cancer families. Cancer Res..

[145] Chen W., Hampel H., Pearlman R., Jones D., Zhao W., Alsomali M., Knight D., Frankel W.L. (2020). Unexpected expression of mismatch repair protein is more commonly seen with pathogenic missense than with other mutations in Lynch syndrome. Hum. Pathol..

[146] Hechtman J.F., Rana S., Middha S., Stadler Z.K., Latham A., Benayed R., Soslow R., Ladanyi M., Yaeger R., Zehir A. (2020). Retained mismatch repair protein expression occurs in approximately 6% of microsatellite instability-high cancers and is associated with missense mutations in mismatch repair genes. Mod. Pathol..

[147] Shia J. (2008). Immunohistochemistry versus microsatellite instability testing for screening colorectal cancer patients at risk for hereditary nonpolyposis colorectal cancer syndrome. Part I. The utility of immunohistochemistry. J. Mol. Diagn..

[148] Van Riel E., Ausems M.G., Hogervorst F.B., Kluijt I., van Gijn M.E., van Echtelt J., Scheidel-Jacobse K., Hennekam E.F., Stulp R.P., Vos Y.J. (2010). A novel pathogenic MLH1 missense mutation, c.112A>C, p.Asn38His, in six families with Lynch syndrome. Hered. Cancer Clin. Pract..

[149] Engel C., Forberg J., Holinski-Feder E., Pagenstecher C., Plaschke J., Kloor M., Poremba C., Pox C.P., Ruschoff J., Keller G. (2006). Novel strategy for optimal sequential application of clinical criteria, immunohistochemistry and microsatellite analysis in the diagnosis of hereditary nonpolyposis colorectal cancer. Int. J. Cancer.

[150] Watson N., Grieu F., Morris M., Harvey J., Stewart C., Schofield L., Goldblatt J., Iacopetta B. (2007). Heterogeneous staining for mismatch repair proteins during population-based prescreening for hereditary nonpolyposis colorectal cancer. J. Mol. Diagn..

[151] Sarode V.R., Robinson L. (2019). Screening for Lynch Syndrome by Immunohistochemistry of Mismatch Repair Proteins: Significance of Indeterminate Result and Correlation With Mutational Studies. Arch. Pathol. Lab. Med..

[152] Bao F., Panarelli N.C., Rennert H., Sherr D.L., Yantiss R.K. (2010). Neoadjuvant therapy induces loss of MSH6 expression in colorectal carcinoma. Am. J. Surg. Pathol..

[153] Kuan S.F., Ren B., Brand R., Dudley B., Pai R.K. (2017). Neoadjuvant therapy in microsatellite-stable colorectal carcinoma induces concomitant loss of MSH6 and Ki-67 expression. Hum. Pathol..

[154] Hissong E., Crowe E.P., Yantiss R.K., Chen Y.T. (2018). Assessing colorectal cancer mismatch repair status in the modern era: A survey of current practices and re-evaluation of the role of microsatellite instability testing. Mod. Pathol..

[155] Lindor N.M., Burgart L.J., Leontovich O., Goldberg R.M., Cunningham J.M., Sargent D.J., Walsh-Vockley C., Petersen G.M., Walsh M.D., Leggett B.A. (2002). Immunohistochemistry versus microsatellite instability testing in phenotyping colorectal tumors. J. Clin. Oncol..

[156] Zhang L. (2008). Immunohistochemistry versus microsatellite instability testing for screening colorectal cancer patients at risk for hereditary nonpolyposis colorectal cancer syndrome. Part II. The utility of microsatellite instability testing. J. Mol. Diagn..

[157] De Jong A.E., Morreau H., Van Puijenbroek M., Eilers P.H., Wijnen J., Nagengast F.M., Griffioen G., Cats A., Menko F.H., Kleibeuker J.H. (2004). The role of mismatch repair gene defects in the development of adenomas in patients with HNPCC. Gastroenterology.

[158] Vasen H.F., den Hartog Jager F.C., Menko F.H., Nagengast F.M. (1989). Screening for hereditary non-polyposis colorectal cancer: A study of 22 kindreds in The Netherlands. Am. J. Med..

[159] Sekine S., Mori T., Ogawa R., Tanaka M., Yoshida H., Taniguchi H., Nakajima T., Sugano K., Yoshida T., Kato M. (2017). Mismatch repair deficiency commonly precedes adenoma formation in Lynch Syndrome-Associated colorectal tumorigenesis. Mod. Pathol..

[160] Ahadova A., Gallon R., Gebert J., Ballhausen A., Endris V., Kirchner M., Stenzinger A., Burn J., von Knebel Doeberitz M., Blaker H. (2018). Three molecular pathways model colorectal carcinogenesis in Lynch syndrome. Int. J. Cancer.

[161] Pino M.S., Mino-Kenudson M., Wildemore B.M., Ganguly A., Batten J., Sperduti I., Iafrate A.J., Chung D.C. (2009). Deficient DNA mismatch repair is common in Lynch syndrome-associated colorectal adenomas. J. Mol. Diagn..

[162] Staffa L., Echterdiek F., Nelius N., Benner A., Werft W., Lahrmann B., Grabe N., Schneider M., Tariverdian M., von Knebel Doeberitz M. (2015). Mismatch repair-deficient crypt foci in Lynch syndrome—Molecular alterations and association with clinical parameters. PLoS ONE.

[163] Kloor M., Huth C., Voigt A.Y., Benner A., Schirmacher P., von Knebel Doeberitz M., Blaker H. (2012). Prevalence of mismatch repair-deficient crypt foci in Lynch syndrome: A pathological study. Lancet Oncol..

[164] Pai R.K., Dudley B., Karloski E., Brand R.E., O’Callaghan N., Rosty C., Buchanan D.D., Jenkins M.A., Thibodeau S.N., French A.J. (2018). DNA mismatch repair protein deficient non-neoplastic colonic crypts: A novel indicator of Lynch syndrome. Mod. Pathol..

[165] Brand R.E., Dudley B., Karloski E., Das R., Fuhrer K., Pai R.K. (2020). Detection of DNA mismatch repair deficient crypts in random colonoscopic biopsies identifies Lynch syndrome patients. Fam. Cancer.

[166] Walsh M.D., Buchanan D.D., Pearson S.A., Clendenning M., Jenkins M.A., Win A.K., Walters R.J., Spring K.J., Nagler B., Pavluk E. (2012). Immunohistochemical testing of conventional adenomas for loss of expression of mismatch repair proteins in Lynch syndrome mutation carriers: A case series from the Australasian site of the colon cancer family registry. Mod. Pathol..

[167] Yurgelun M.B., Goel A., Hornick J.L., Sen A., Turgeon D.K., Ruffin M.T., Marcon N.E., Baron J.A., Bresalier R.S., Syngal S. (2012). Microsatellite instability and DNA mismatch repair protein deficiency in Lynch syndrome colorectal polyps. Cancer Prev. Res..

[168] Balmana J., Stockwell D.H., Steyerberg E.W., Stoffel E.M., Deffenbaugh A.M., Reid J.E., Ward B., Scholl T., Hendrickson B., Tazelaar J. (2006). Prediction of MLH1 and MSH2 mutations in Lynch syndrome. JAMA.

[169] Chen S., Wang W., Lee S., Nafa K., Lee J., Romans K., Watson P., Gruber S.B., Euhus D., Kinzler K.W. (2006). Prediction of germline mutations and cancer risk in the Lynch syndrome. JAMA.

[170] Tresallet C., Brouquet A., Julie C., Beauchet A., Vallot C., Menegaux F., Mitry E., Radvanyi F., Malafosse R., Rougier P. (2012). Evaluation of predictive models in daily practice for the identification of patients with Lynch syndrome. Int. J. Cancer.

[171] Peltomaki P. (2016). Update on Lynch syndrome genomics. Fam. Cancer.

[172] Heinen C.D., Juel Rasmussen L. (2012). Determining the functional significance of mismatch repair gene missense variants using biochemical and cellular assays. Hered. Cancer Clin. Pract..

[173] Gazzoli I., Loda M., Garber J., Syngal S., Kolodner R.D. (2002). A hereditary nonpolyposis colorectal carcinoma case associated with hypermethylation of the MLH1 gene in normal tissue and loss of heterozygosity of the unmethylated allele in the resulting microsatellite instability-high tumor. Cancer Res..

[174] Chan T.L., Yuen S.T., Kong C.K., Chan Y.W., Chan A.S., Ng W.F., Tsui W.Y., Lo M.W., Tam W.Y., Li V.S. (2006). Heritable germline epimutation of MSH2 in a family with hereditary nonpolyposis colorectal cancer. Nat. Genet..

[175] Hitchins M.P. (2013). The role of epigenetics in Lynch syndrome. Fam. Cancer.

[176] Pritchard C.C., Smith C., Salipante S.J., Lee M.K., Thornton A.M., Nord A.S., Gulden C., Kupfer S.S., Swisher E.M., Bennett R.L. (2012). ColoSeq provides comprehensive lynch and polyposis syndrome mutational analysis using massively parallel sequencing. J. Mol. Diagn..

[177] Clendenning M., Hampel H., LaJeunesse J., Lindblom A., Lockman J., Nilbert M., Senter L., Sotamaa K., de la Chapelle A. (2006). Long-range PCR facilitates the identification of PMS2-specific mutations. Hum. Mutat..

[178] Vaughn C.P., Robles J., Swensen J.J., Miller C.E., Lyon E., Mao R., Bayrak-Toydemir P., Samowitz W.S. (2010). Clinical analysis of PMS2: Mutation detection and avoidance of pseudogenes. Hum. Mutat..

[179] Vaughn C.P., Baker C.L., Samowitz W.S., Swensen J.J. (2013). The frequency of previously undetectable deletions involving 3’ Exons of the PMS2 gene. Genes Chromosomes Cancer.

[180] Wimmer K., Wernstedt A. (2014). PMS2 gene mutational analysis: Direct cDNA sequencing to circumvent pseudogene interference. Methods Mol. Biol..

[181] Li J., Dai H., Feng Y., Tang J., Chen S., Tian X., Gorman E., Schmitt E.S., Hansen T.A., Wang J. (2015). A Comprehensive Strategy for Accurate Mutation Detection of the Highly Homologous PMS2. J. Mol. Diagn..

[182] Gould G.M., Grauman P.V., Theilmann M.R., Spurka L., Wang I.E., Melroy L.M., Chin R.G., Hite D.H., Chu C.S., Maguire J.R. (2018). Detecting clinically actionable variants in the 3′ exons of PMS2 via a reflex workflow based on equivalent hybrid capture of the gene and its pseudogene. BMC Med. Genet..

[183] Antelo M., Golubicki M., Roca E., Mendez G., Carballido M., Iseas S., Cuatrecasas M., Moreira L., Sanchez A., Carballal S. (2019). Lynch-like syndrome is as frequent as Lynch syndrome in early-onset nonfamilial nonpolyposis colorectal cancer. Int. J. Cancer.

[184] Ligtenberg M.J., Kuiper R.P., Chan T.L., Goossens M., Hebeda K.M., Voorendt M., Lee T.Y., Bodmer D., Hoenselaar E., Hendriks-Cornelissen S.J. (2009). Heritable somatic methylation and inactivation of MSH2 in families with Lynch syndrome due to deletion of the 3′ exons of TACSTD1. Nat. Genet..

[185] Rumilla K., Schowalter K.V., Lindor N.M., Thomas B.C., Mensink K.A., Gallinger S., Holter S., Newcomb P.A., Potter J.D., Jenkins M.A. (2011). Frequency of deletions of EPCAM (TACSTD1) in MSH2-associated Lynch syndrome cases. J. Mol. Diagn..

[186] Kuiper R.P., Vissers L.E., Venkatachalam R., Bodmer D., Hoenselaar E., Goossens M., Haufe A., Kamping E., Niessen R.C., Hogervorst F.B. (2011). Recurrence and variability of germline EPCAM deletions in Lynch syndrome. Hum. Mutat..

[187] Hitchins M.P. (2016). Finding the needle in a haystack: Identification of cases of Lynch syndrome with MLH1 epimutation. Fam. Cancer.

[188] Leclerc J., Flament C., Lovecchio T., Delattre L., Ait Yahya E., Baert-Desurmont S., Burnichon N., Bronner M., Cabaret O., Lejeune S. (2018). Diversity of genetic events associated with MLH1 promoter methylation in Lynch syndrome families with heritable constitutional epimutation. Genet. Med..

[189] Damaso E., Canet-Hermida J., Vargas-Parra G., Velasco A., Marin F., Darder E., Del Valle J., Fernandez A., Izquierdo A., Mateu G. (2019). Highly sensitive MLH1 methylation analysis in blood identifies a cancer patient with low-level mosaic MLH1 epimutation. Clin. Epigenetics.

[190] Arnold A.M., Morak M., Benet-Pages A., Laner A., Frishman D., Holinski-Feder E. (2020). Targeted deep-intronic sequencing in a cohort of unexplained cases of suspected Lynch syndrome. Eur. J. Hum. Genet..

[191] Clendenning M., Buchanan D.D., Walsh M.D., Nagler B., Rosty C., Thompson B., Spurdle A.B., Hopper J.L., Jenkins M.A., Young J.P. (2011). Mutation deep within an intron of MSH2 causes Lynch syndrome. Fam. Cancer.

[192] Casadei S., Gulsuner S., Shirts B.H., Mandell J.B., Kortbawi H.M., Norquist B.S., Swisher E.M., Lee M.K., Goldberg Y., O′Connor R. (2019). Characterization of splice-altering mutations in inherited predisposition to cancer. Proc. Natl. Acad. Sci. USA.

[193] Morak M., Koehler U., Schackert H.K., Steinke V., Royer-Pokora B., Schulmann K., Kloor M., Hochter W., Weingart J., Keiling C. (2011). Biallelic MLH1 SNP cDNA expression or constitutional promoter methylation can hide genomic rearrangements causing Lynch syndrome. J. Med. Genet..

[194] Wagner A., van der Klift H., Franken P., Wijnen J., Breukel C., Bezrookove V., Smits R., Kinarsky Y., Barrows A., Franklin B. (2002). A 10-Mb paracentric inversion of chromosome arm 2p inactivates MSH2 and is responsible for hereditary nonpolyposis colorectal cancer in a North-American kindred. Genes Chromosomes Cancer.

[195] Chen J.M. (2008). The 10-Mb paracentric inversion of chromosome arm 2p in activating MSH2 and causing hereditary nonpolyposis colorectal cancer: Re-annotation and mutational mechanisms. Genes Chromosomes Cancer.

[196] Liu Q., Hesson L.B., Nunez A.C., Packham D., Williams R., Ward R.L., Sloane M.A. (2016). A cryptic paracentric inversion of MSH2 exons 2-6 causes Lynch syndrome. Carcinogenesis.

[197] Haraldsdottir S., Rafnar T., Frankel W.L., Einarsdottir S., Sigurdsson A., Hampel H., Snaebjornsson P., Masson G., Weng D., Arngrimsson R. (2017). Comprehensive population-wide analysis of Lynch syndrome in Iceland reveals founder mutations in MSH6 and PMS2. Nat. Commun..

[198] Van der Klift H.M., Tops C.M., Hes F.J., Devilee P., Wijnen J.T. (2012). Insertion of an SVA element, a nonautonomous retrotransposon, in PMS2 intron 7 as a novel cause of Lynch syndrome. Hum. Mutat..

[199] Morak M., Steinke-Lange V., Massdorf T., Benet-Pages A., Locher M., Laner A., Kayser K., Aretz S., Holinski-Feder E. (2020). Prevalence of CNV-neutral structural genomic rearrangements in MLH1, MSH2, and PMS2 not detectable in routine NGS diagnostics. Fam. Cancer.

[200] Morak M., Schaefer K., Steinke-Lange V., Koehler U., Keinath S., Massdorf T., Mauracher B., Rahner N., Bailey J., Kling C. (2019). Full-length transcript amplification and sequencing as universal method to test mRNA integrity and biallelic expression in mismatch repair genes. Eur. J. Hum. Genet..

[201] Rhees J., Arnold M., Boland C.R. (2014). Inversion of exons 1-7 of the MSH2 gene is a frequent cause of unexplained Lynch syndrome in one local population. Fam. Cancer.

[202] Kloor M., Sutter C., Wentzensen N., Cremer F.W., Buckowitz A., Keller M., von Knebel Doeberitz M., Gebert J. (2004). A large MSH2 Alu insertion mutation causes HNPCC in a German kindred. Hum. Genet..

[203] Solassol J., Larrieux M., Leclerc J., Ducros V., Corsini C., Chiesa J., Pujol P., Rey J.M. (2019). Alu element insertion in the MLH1 exon 6 coding sequence as a mutation predisposing to Lynch syndrome. Hum. Mutat..

[204] Pastrello C., Fornasarig M., Pin E., Berto E., Pivetta B., Viel A. (2009). Somatic mosaicism in a patient with Lynch syndrome. Am. J. Med. Genet. A.

[205] Sourrouille I., Coulet F., Lefevre J.H., Colas C., Eyries M., Svrcek M., Bardier-Dupas A., Parc Y., Soubrier F. (2013). Somatic mosaicism and double somatic hits can lead to MSI colorectal tumors. Fam. Cancer.

[206] Geurts-Giele W.R., Rosenberg E.H., Rens A.V., Leerdam M.E.V., Dinjens W.N., Bleeker F.E. (2019). Somatic mosaicism by a de novo MLH1 mutation as a cause of Lynch syndrome. Mol. Genet. Genom. Med..

[207] Jansen A.M.L., Goel A. (2020). Mosaicism in Patients With Colorectal Cancer or Polyposis Syndromes: A Systematic Review. Clin. Gastroenterol. Hepatol..

[208] Sutcliffe E.G., Bartenbaker Thompson A., Stettner A.R., Marshall M.L., Roberts M.E., Susswein L.R., Wang Y., Klein R.T., Hruska K.S., Solomon B.D. (2019). Multi-gene panel testing confirms phenotypic variability in MUTYH-Associated Polyposis. Fam. Cancer.

[209] Morak M., Heidenreich B., Keller G., Hampel H., Laner A., de la Chapelle A., Holinski-Feder E. (2014). Biallelic MUTYH mutations can mimic Lynch syndrome. Eur. J. Hum. Genet..

[210] Pearlman R., Frankel W.L., Swanson B., Zhao W., Yilmaz A., Miller K., Bacher J., Bigley C., Nelsen L., Goodfellow P.J. (2017). Prevalence and Spectrum of Germline Cancer Susceptibility Gene Mutations Among Patients With Early-Onset Colorectal Cancer. JAMA Oncol..

[211] Lefevre J.H., Colas C., Coulet F., Bonilla C., Mourra N., Flejou J.F., Tiret E., Bodmer W., Soubrier F., Parc Y. (2010). MYH biallelic mutation can inactivate the two genetic pathways of colorectal cancer by APC or MLH1 transversions. Fam. Cancer.

[212] Palles C., Cazier J.B., Howarth K.M., Domingo E., Jones A.M., Broderick P., Kemp Z., Spain S.L., Guarino E., Salguero I. (2013). Germline mutations affecting the proofreading domains of POLE and POLD1 predispose to colorectal adenomas and carcinomas. Nat. Genet..

[213] Elsayed F.A., Kets C.M., Ruano D., van den Akker B., Mensenkamp A.R., Schrumpf M., Nielsen M., Wijnen J.T., Tops C.M., Ligtenberg M.J. (2015). Germline variants in POLE are associated with early onset mismatch repair deficient colorectal cancer. Eur. J. Hum. Genet..

[214] Adam R., Spier I., Zhao B., Kloth M., Marquez J., Hinrichsen I., Kirfel J., Tafazzoli A., Horpaopan S., Uhlhaas S. (2016). Exome Sequencing Identifies Biallelic MSH3 Germline Mutations as a Recessive Subtype of Colorectal Adenomatous Polyposis. Am. J. Hum. Genet..

[215] Damaso E., Gonzalez-Acosta M., Vargas-Parra G., Navarro M., Balmana J., Ramon Y.C.T., Tuset N., Thompson B.A., Marin F., Fernandez A. (2020). Comprehensive Constitutional Genetic and Epigenetic Characterization of Lynch-Like Individuals. Cancers.

[216] Xavier A., Olsen M.F., Lavik L.A., Johansen J., Singh A.K., Sjursen W., Scott R.J., Talseth-Palmer B.A. (2019). Comprehensive mismatch repair gene panel identifies variants in patients with Lynch-like syndrome. Mol. Genet. Genom. Med..

[217] Vargas-Parra G.M., Gonzalez-Acosta M., Thompson B.A., Gomez C., Fernandez A., Damaso E., Pons T., Morak M., Del Valle J., Iglesias S. (2017). Elucidating the molecular basis of MSH2-deficient tumors by combined germline and somatic analysis. Int. J. Cancer.

[218] Xicola R.M., Clark J.R., Carroll T., Alvikas J., Marwaha P., Regan M.R., Lopez-Giraldez F., Choi J., Emmadi R., Alagiozian-Angelova V. (2019). Implication of DNA repair genes in Lynch-like syndrome. Fam. Cancer.

[219] Golubicki M., Bonjoch L., Acuna-Ochoa J.G., Diaz-Gay M., Munoz J., Cuatrecasas M., Ocana T., Iseas S., Mendez G., Cisterna D. (2020). Germline biallelic Mcm8 variants are associated with early-onset Lynch-like syndrome. JCI Insight.

[220] Vos J.R., Fakkert I.E., Spruijt L., Willems R.W., Langenveld S., Mensenkamp A.R., Leter E.M., Nagtegaal I.D., Ligtenberg M.J.L., Hoogerbrugge N. (2020). Evaluation of yield and experiences of age-related molecular investigation for heritable and nonheritable causes of mismatch repair deficient colorectal cancer to identify Lynch syndrome. Int. J. Cancer.

[221] Jansen A.M., van Wezel T., van den Akker B.E., Ventayol Garcia M., Ruano D., Tops C.M., Wagner A., Letteboer T.G., Gomez-Garcia E.B., Devilee P. (2016). Combined mismatch repair and POLE/POLD1 defects explain unresolved suspected Lynch syndrome cancers. Eur. J. Hum. Genet..

[222] Porkka N., Lahtinen L., Ahtiainen M., Bohm J.P., Kuopio T., Eldfors S., Mecklin J.P., Seppala T.T., Peltomaki P. (2019). Epidemiological, clinical and molecular characterization of Lynch-like syndrome: A population-based study. Int. J. Cancer.

[223] Mensenkamp A.R., Vogelaar I.P., van Zelst-Stams W.A., Goossens M., Ouchene H., Hendriks-Cornelissen S.J., Kwint M.P., Hoogerbrugge N., Nagtegaal I.D., Ligtenberg M.J. (2014). Somatic mutations in MLH1 and MSH2 are a frequent cause of mismatch-repair deficiency in Lynch syndrome-like tumors. Gastroenterology.

[224] Geurts-Giele W.R., Leenen C.H., Dubbink H.J., Meijssen I.C., Post E., Sleddens H.F., Kuipers E.J., Goverde A., van den Ouweland A.M., van Lier M.G. (2014). Somatic aberrations of mismatch repair genes as a cause of microsatellite-unstable cancers. J. Pathol..

[225] Haraldsdottir S., Hampel H., Tomsic J., Frankel W.L., Pearlman R., de la Chapelle A., Pritchard C.C. (2014). Colon and endometrial cancers with mismatch repair deficiency can arise from somatic, rather than germline, mutations. Gastroenterology.

[226] Billingsley C.C., Cohn D.E., Mutch D.G., Stephens J.A., Suarez A.A., Goodfellow P.J. (2015). Polymerase varepsilon (POLE) mutations in endometrial cancer: Clinical outcomes and implications for Lynch syndrome testing. Cancer.

[227] Joly M.O., Attignon V., Saurin J.C., Desseigne F., Leroux D., Martin-Denavit T., Giraud S., Bonnet-Dupeyron M.N., Faivre L., Auclair J. (2015). Somatic MMR gene mutations as a cause for MSI-H sebaceous neoplasms in Muir-Torre syndrome-like patients. Hum. Mutat..

[228] Ullman T.A., Itzkowitz S.H. (2011). Intestinal inflammation and cancer. Gastroenterology.

[229] Rigter L.S., Snaebjornsson P., Rosenberg E.H., Atmodimedjo P.N., Aleman B.M., Ten Hoeve J., Geurts-Giele W.R., van Ravesteyn T.W., Hoeksel J., Meijer G.A. (2018). Double somatic mutations in mismatch repair genes are frequent in colorectal cancer after Hodgkin′s lymphoma treatment. Gut.

[230] Li F., Mao G., Tong D., Huang J., Gu L., Yang W., Li G.M. (2013). The histone mark H3K36me3 regulates human DNA mismatch repair through its interaction with MutSalpha. Cell.

[231] Ortega J., Li J.Y., Lee S., Tong D., Gu L., Li G.M. (2015). Phosphorylation of PCNA by EGFR inhibits mismatch repair and promotes misincorporation during DNA synthesis. Proc. Natl. Acad. Sci. USA.

[232] Buchanan D.D., Rosty C., Clendenning M., Spurdle A.B., Win A.K. (2014). Clinical problems of colorectal cancer and endometrial cancer cases with unknown cause of tumor mismatch repair deficiency (suspected Lynch syndrome). Appl. Clin. Genet..

[233] Steinke V., Holzapfel S., Loeffler M., Holinski-Feder E., Morak M., Schackert H.K., Gorgens H., Pox C., Royer-Pokora B., von Knebel-Doeberitz M. (2014). Evaluating the performance of clinical criteria for predicting mismatch repair gene mutations in Lynch syndrome: A comprehensive analysis of 3671 families. Int. J. Cancer.

[234] Buchanan D.D., Clendenning M., Rosty C., Eriksen S.V., Walsh M.D., Walters R.J., Thibodeau S.N., Stewart J., Preston S., Win A.K. (2017). Tumor testing to identify lynch syndrome in two Australian colorectal cancer cohorts. J. Gastroenterol. Hepatol..

[235] Mills A.M., Sloan E.A., Thomas M., Modesitt S.C., Stoler M.H., Atkins K.A., Moskaluk C.A. (2016). Clinicopathologic Comparison of Lynch Syndrome-associated and “Lynch-like” Endometrial Carcinomas Identified on Universal Screening Using Mismatch Repair Protein Immunohistochemistry. Am. J. Surg. Pathol..

[236] Kang S.Y., Park C.K., Chang D.K., Kim J.W., Son H.J., Cho Y.B., Yun S.H., Kim H.C., Kwon M., Kim K.M. (2015). Lynch-like syndrome: Characterization and comparison with EPCAM deletion carriers. Int. J. Cancer.

[237] Rodriguez-Soler M., Perez-Carbonell L., Guarinos C., Zapater P., Castillejo A., Barbera V.M., Juarez M., Bessa X., Xicola R.M., Clofent J. (2013). Risk of cancer in cases of suspected lynch syndrome without germline mutation. Gastroenterology.

[238] Win A.K., Buchanan D.D., Rosty C., MacInnis R.J., Dowty J.G., Dite G.S., Giles G.G., Southey M.C., Young J.P., Clendenning M. (2015). Role of tumour molecular and pathology features to estimate colorectal cancer risk for first-degree relatives. Gut.

[239] Dempsey K.M., Broaddus R., You Y.N., Noblin S.J., Mork M., Fellman B., Urbauer D., Daniels M., Lu K. (2015). Is it all Lynch syndrome?: An assessment of family history in individuals with mismatch repair-deficient tumors. Genet. Med..

[240] Kato A., Sato N., Sugawara T., Takahashi K., Kito M., Makino K., Sato T., Shimizu D., Shirasawa H., Miura H. (2016). Isolated Loss of PMS2 Immunohistochemical Expression is Frequently Caused by Heterogenous MLH1 Promoter Hypermethylation in Lynch Syndrome Screening for Endometrial Cancer Patients. Am. J. Surg. Pathol..

[241] Newton K., Jorgensen N.M., Wallace A.J., Buchanan D.D., Lalloo F., McMahon R.F., Hill J., Evans D.G. (2014). Tumour MLH1 promoter region methylation testing is an effective prescreen for Lynch Syndrome (HNPCC). J. Med. Genet..

[242] Parsons M.T., Buchanan D.D., Thompson B., Young J.P., Spurdle A.B. (2012). Correlation of tumour BRAF mutations and MLH1 methylation with germline mismatch repair (MMR) gene mutation status: A literature review assessing utility of tumour features for MMR variant classification. J. Med. Genet..

[243] Lu K.H., Schorge J.O., Rodabaugh K.J., Daniels M.S., Sun C.C., Soliman P.T., White K.G., Luthra R., Gershenson D.M., Broaddus R.R. (2007). Prospective determination of prevalence of lynch syndrome in young women with endometrial cancer. J. Clin. Oncol..

[244] Roberts M.E., Riegert-Johnson D.L., Thomas B.C., Rumilla K.M., Thomas C.S., Heckman M.G., Purcell J.U., Hanson N.B., Leppig K.A., Lim J. (2014). A clinical scoring system to identify patients with sebaceous neoplasms at risk for the Muir-Torre variant of Lynch syndrome. Genet. Med..

[245] Shannon C., Kirk J., Barnetson R., Evans J., Schnitzler M., Quinn M., Hacker N., Crandon A., Harnett P. (2003). Incidence of microsatellite instability in synchronous tumors of the ovary and endometrium. Clin. Cancer Res..

[246] Soliman P.T., Broaddus R.R., Schmeler K.M., Daniels M.S., Gonzalez D., Slomovitz B.M., Gershenson D.M., Lu K.H. (2005). Women with synchronous primary cancers of the endometrium and ovary: Do they have Lynch syndrome?. J. Clin. Oncol..

[247] Wagner A., Hendriks Y., Meijers-Heijboer E.J., de Leeuw W.J., Morreau H., Hofstra R., Tops C., Bik E., Brocker-Vriends A.H., van Der Meer C. (2001). Atypical HNPCC owing to MSH6 germline mutations: Analysis of a large Dutch pedigree. J. Med. Genet..

[248] Pal T., Permuth-Wey J., Sellers T.A. (2008). A review of the clinical relevance of mismatch-repair deficiency in ovarian cancer. Cancer.

[249] Murphy M.A., Wentzensen N. (2011). Frequency of mismatch repair deficiency in ovarian cancer: A systematic review This article is a US Government work and, as such, is in the public domain of the United States of America. Int. J. Cancer.

[250] Joost P., Veurink N., Holck S., Klarskov L., Bojesen A., Harbo M., Baldetorp B., Rambech E., Nilbert M. (2014). Heterogenous mismatch-repair status in colorectal cancer. Diagn. Pathol..

[251] Pai R.K., Plesec T.P., Abdul-Karim F.W., Yang B., Marquard J., Shadrach B., Roma A.R. (2015). Abrupt loss of MLH1 and PMS2 expression in endometrial carcinoma: Molecular and morphologic analysis of 6 cases. Am. J. Surg. Pathol..

[252] Weisenberger D.J., Siegmund K.D., Campan M., Young J., Long T.I., Faasse M.A., Kang G.H., Widschwendter M., Weener D., Buchanan D. (2006). CpG island methylator phenotype underlies sporadic microsatellite instability and is tightly associated with BRAF mutation in colorectal cancer. Nat. Genet..

[253] Toyota M., Ahuja N., Ohe-Toyota M., Herman J.G., Baylin S.B., Issa J.P. (1999). CpG island methylator phenotype in colorectal cancer. Proc. Natl. Acad. Sci. USA.

[254] Deng G., Chen A., Hong J., Chae H.S., Kim Y.S. (1999). Methylation of CpG in a small region of the hMLH1 promoter invariably correlates with the absence of gene expression. Cancer Res..

[255] Capel E., Flejou J.F., Hamelin R. (2007). Assessment of MLH1 promoter methylation in relation to gene expression requires specific analysis. Oncogene.

[256] Ruemmele P., Dietmaier W., Terracciano L., Tornillo L., Bataille F., Kaiser A., Wuensch P.H., Heinmoeller E., Homayounfar K., Luettges J. (2009). Histopathologic features and microsatellite instability of cancers of the papilla of vater and their precursor lesions. Am. J. Surg. Pathol..

[257] Bennett J.A., Pesci A., Morales-Oyarvide V., Da Silva A., Nardi V., Oliva E. (2019). Incidence of Mismatch Repair Protein Deficiency and Associated Clinicopathologic Features in a Cohort of 104 Ovarian Endometrioid Carcinomas. Am. J. Surg. Pathol..

[258] Jones J.C., Renfro L.A., Al-Shamsi H.O., Schrock A.B., Rankin A., Zhang B.Y., Kasi P.M., Voss J.S., Leal A.D., Sun J. (2017). (Non-V600) BRAF Mutations Define a Clinically Distinct Molecular Subtype of Metastatic Colorectal Cancer. J. Clin. Oncol..

[259] Rad R., Cadinanos J., Rad L., Varela I., Strong A., Kriegl L., Constantino-Casas F., Eser S., Hieber M., Seidler B. (2013). A genetic progression model of Braf(V600E)-induced intestinal tumorigenesis reveals targets for therapeutic intervention. Cancer Cell.

[260] Sakamoto N., Feng Y., Stolfi C., Kurosu Y., Green M., Lin J., Green M.E., Sentani K., Yasui W., McMahon M. (2017). BRAF(V600E) cooperates with CDX2 inactivation to promote serrated colorectal tumorigenesis. Elife.

[261] Bond C.E., Liu C., Kawamata F., McKeone D.M., Fernando W., Jamieson S., Pearson S.A., Kane A., Woods S.L., Lannagan T.R.M. (2018). Oncogenic BRAF mutation induces DNA methylation changes in a murine model for human serrated colorectal neoplasia. Epigenetics.

[262] Wang L., Cunningham J.M., Winters J.L., Guenther J.C., French A.J., Boardman L.A., Burgart L.J., McDonnell S.K., Schaid D.J., Thibodeau S.N. (2003). BRAF mutations in colon cancer are not likely attributable to defective DNA mismatch repair. Cancer Res..

[263] Bouzourene H., Hutter P., Losi L., Martin P., Benhattar J. (2010). Selection of patients with germline MLH1 mutated Lynch syndrome by determination of MLH1 methylation and BRAF mutation. Fam. Cancer.

[264] Domingo E., Laiho P., Ollikainen M., Pinto M., Wang L., French A.J., Westra J., Frebourg T., Espin E., Armengol M. (2004). BRAF screening as a low-cost effective strategy for simplifying HNPCC genetic testing. J. Med. Genet..

[265] Loughrey M.B., Waring P.M., Tan A., Trivett M., Kovalenko S., Beshay V., Young M.A., McArthur G., Boussioutas A., Dobrovic A. (2007). Incorporation of somatic BRAF mutation testing into an algorithm for the investigation of hereditary non-polyposis colorectal cancer. Fam. Cancer.

[266] Bessa X., Balleste B., Andreu M., Castells A., Bellosillo B., Balaguer F., Castellvi-Bel S., Paya A., Jover R., Alenda C. (2008). A prospective, multicenter, population-based study of BRAF mutational analysis for Lynch syndrome screening. Clin. Gastroenterol. Hepatol..

[267] Moreira L., Munoz J., Cuatrecasas M., Quintanilla I., Leoz M.L., Carballal S., Ocana T., Lopez-Ceron M., Pellise M., Castellvi-Bel S. (2015). Prevalence of somatic mutl homolog 1 promoter hypermethylation in Lynch syndrome colorectal cancer. Cancer.

[268] Dankner M., Rose A.A.N., Rajkumar S., Siegel P.M., Watson I.R. (2018). Classifying BRAF alterations in cancer: New rational therapeutic strategies for actionable mutations. Oncogene.

[269] Mutch D.G., Powell M.A., Mallon M.A., Goodfellow P.J. (2004). RAS/RAF mutation and defective DNA mismatch repair in endometrial cancers. Am. J. Obs. Gynecol..

[270] Salvesen H.B., Kumar R., Stefansson I., Angelini S., MacDonald N., Smeds J., Jacobs I.J., Hemminki K., Das S., Akslen L.A. (2005). Low frequency of BRAF and CDKN2A mutations in endometrial cancer. Int. J. Cancer.

[271] Kawaguchi M., Yanokura M., Banno K., Kobayashi Y., Kuwabara Y., Kobayashi M., Nomura H., Hirasawa A., Susumu N., Aoki D. (2009). Analysis of a correlation between the BRAF V600E mutation and abnormal DNA mismatch repair in patients with sporadic endometrial cancer. Int. J. Oncol..

[272] Peterson L.M., Kipp B.R., Halling K.C., Kerr S.E., Smith D.I., Distad T.J., Clayton A.C., Medeiros F. (2012). Molecular characterization of endometrial cancer: A correlative study assessing microsatellite instability, MLH1 hypermethylation, DNA mismatch repair protein expression, and PTEN, PIK3CA, KRAS, and BRAF mutation analysis. Int. J. Gynecol. Pathol..

[273] Feng Y.Z., Shiozawa T., Miyamoto T., Kashima H., Kurai M., Suzuki A., Konishi I. (2005). BRAF mutation in endometrial carcinoma and hyperplasia: Correlation with KRAS and p53 mutations and mismatch repair protein expression. Clin. Cancer Res..

[274] Schrock A.B., Devoe C.E., McWilliams R., Sun J., Aparicio T., Stephens P.J., Ross J.S., Wilson R., Miller V.A., Ali S.M. (2017). Genomic Profiling of Small-Bowel Adenocarcinoma. JAMA Oncol..

[275] Cornejo K.M., Hutchinson L., Deng A., Tomaszewicz K., Welch M., Lyle S., Dresser K., Cosar E.F. (2014). BRAF/KRAS gene sequencing of sebaceous neoplasms after mismatch repair protein analysis. Hum. Pathol..

[276] Capper D., Voigt A., Bozukova G., Ahadova A., Kickingereder P., von Deimling A., von Knebel Doeberitz M., Kloor M. (2013). BRAF V600E-specific immunohistochemistry for the exclusion of Lynch syndrome in MSI-H colorectal cancer. Int. J. Cancer.

[277] Thiel A., Heinonen M., Kantonen J., Gylling A., Lahtinen L., Korhonen M., Kytola S., Mecklin J.P., Orpana A., Peltomaki P. (2013). BRAF mutation in sporadic colorectal cancer and Lynch syndrome. Virchows Arch..

[278] Toon C.W., Walsh M.D., Chou A., Capper D., Clarkson A., Sioson L., Clarke S., Mead S., Walters R.J., Clendenning M. (2013). BRAFV600E immunohistochemistry facilitates universal screening of colorectal cancers for Lynch syndrome. Am. J. Surg. Pathol..

[279] Lasota J., Kowalik A., Wasag B., Wang Z.F., Felisiak-Golabek A., Coates T., Kopczynski J., Gozdz S., Miettinen M. (2014). Detection of the BRAF V600E mutation in colon carcinoma: Critical evaluation of the imunohistochemical approach. Am. J. Surg. Pathol..

[280] Adackapara C.A., Sholl L.M., Barletta J.A., Hornick J.L. (2013). Immunohistochemistry using the BRAF V600E mutation-specific monoclonal antibody VE1 is not a useful surrogate for genotyping in colorectal adenocarcinoma. Histopathology.

[281] Perez-Carbonell L., Alenda C., Paya A., Castillejo A., Barbera V.M., Guillen C., Rojas E., Acame N., Gutierrez-Avino F.J., Castells A. (2010). Methylation analysis of MLH1 improves the selection of patients for genetic testing in Lynch syndrome. J. Mol. Diagn..

[282] Gausachs M., Mur P., Corral J., Pineda M., Gonzalez S., Benito L., Menendez M., Espinas J.A., Brunet J., Iniesta M.D. (2012). MLH1 promoter hypermethylation in the analytical algorithm of Lynch syndrome: A cost-effectiveness study. Eur. J. Hum. Genet..

[283] Adar T., Rodgers L.H., Shannon K.M., Yoshida M., Ma T., Mattia A., Lauwers G.Y., Iafrate A.J., Chung D.C. (2017). A tailored approach to BRAF and MLH1 methylation testing in a universal screening program for Lynch syndrome. Mod. Pathol..

[284] Rahner N., Friedrichs N., Steinke V., Aretz S., Friedl W., Buettner R., Mangold E., Propping P., Walldorf C. (2008). Coexisting somatic promoter hypermethylation and pathogenic MLH1 germline mutation in Lynch syndrome. J. Pathol..

[285] Yokoyama T., Takehara K., Sugimoto N., Kaneko K., Fujimoto E., Okazawa-Sakai M., Okame S., Shiroyama Y., Teramoto N., Ohsumi S. (2018). Lynch syndrome-associated endometrial carcinoma with MLH1 germline mutation and MLH1 promoter hypermethylation: A case report and literature review. BMC Cancer.

[286] Bettstetter M., Dechant S., Ruemmele P., Grabowski M., Keller G., Holinski-Feder E., Hartmann A., Hofstaedter F., Dietmaier W. (2007). Distinction of hereditary nonpolyposis colorectal cancer and sporadic microsatellite-unstable colorectal cancer through quantification of MLH1 methylation by real-time PCR. Clin. Cancer Res..

[287] Herman J.G., Merlo A., Mao L., Lapidus R.G., Issa J.P., Davidson N.E., Sidransky D., Baylin S.B. (1995). Inactivation of the CDKN2/p16/MTS1 gene is frequently associated with aberrant DNA methylation in all common human cancers. Cancer Res..

[288] Ahuja N., Mohan A.L., Li Q., Stolker J.M., Herman J.G., Hamilton S.R., Baylin S.B., Issa J.P. (1997). Association between CpG island methylation and microsatellite instability in colorectal cancer. Cancer Res..

[289] Paya A., Alenda C., Perez-Carbonell L., Rojas E., Soto J.L., Guillen C., Castillejo A., Barbera V.M., Carrato A., Castells A. (2009). Utility of p16 immunohistochemistry for the identification of Lynch syndrome. Clin. Cancer Res..

[290] Boissiere-Michot F., Frugier H., Ho-Pun-Cheung A., Lopez-Crapez E., Duffour J., Bibeau F. (2016). Immunohistochemical staining for p16 and BRAFV600E is useful to distinguish between sporadic and hereditary (Lynch syndrome-related) microsatellite instable colorectal carcinomas. Virchows Arch..

[291] Kim J.H., Kim K.J., Rhee Y.Y., Bae J.M., Cho N.Y., Lee H.S., Kang G.H. (2015). Gastric-type expression signature in serrated pathway-associated colorectal tumors. Hum. Pathol..

[292] Pai R.K., Shadrach B.L., Carver P., Heald B., Moline J., Church J., Kalady M.F., Burke C.A., Plesec T.P., Lai K.K. (2014). Immunohistochemistry for annexin A10 can distinguish sporadic from Lynch syndrome-associated microsatellite-unstable colorectal carcinoma. Am. J. Surg. Pathol..

[293] Cohen S.A., Turner E.H., Beightol M.B., Jacobson A., Gooley T.A., Salipante S.J., Haraldsdottir S., Smith C., Scroggins S., Tait J.F. (2016). Frequent PIK3CA Mutations in Colorectal and Endometrial Tumors with 2 or More Somatic Mutations in Mismatch Repair Genes. Gastroenterology.

[294] Alexandrov L.B., Nik-Zainal S., Wedge D.C., Aparicio S.A., Behjati S., Biankin A.V., Bignell G.R., Bolli N., Borg A., Borresen-Dale A.L. (2013). Signatures of mutational processes in human cancer. Nature.

[295] Alexandrov L.B., Ju Y.S., Haase K., Van Loo P., Martincorena I., Nik-Zainal S., Totoki Y., Fujimoto A., Nakagawa H., Shibata T. (2016). Mutational signatures associated with tobacco smoking in human cancer. Science.

[296] Georgeson P., Walsh M.D., Clendenning M., Daneshvar S., Pope B.J., Mahmood K., Joo J.E., Jayasekara H., Jenkins M.A., Winship I.M. (2019). Tumor mutational signatures in sebaceous skin lesions from individuals with Lynch syndrome. Mol. Genet. Genom. Med..

[297] Hampel H., Pearlman R., Beightol M., Zhao W., Jones D., Frankel W.L., Goodfellow P.J., Yilmaz A., Miller K., Bacher J. (2018). Assessment of Tumor Sequencing as a Replacement for Lynch Syndrome Screening and Current Molecular Tests for Patients With Colorectal Cancer. JAMA Oncol..

